# Role of the Psi Packaging Signal and Dimerization Initiation Sequence in the Organization of Rous Sarcoma Virus Gag-gRNA Co-Condensates

**DOI:** 10.3390/v17010097

**Published:** 2025-01-13

**Authors:** Gregory S. Lambert, Rebecca J. Kaddis Maldonado, Leslie J. Parent

**Affiliations:** 1 Department of Medicine, Penn State College of Medicine, 500 University Drive, Hershey, PA 17033, USA; glambert@pennstatehealth.psu.edu (G.S.L.); rkaddis@pennstatehealth.psu.edu (R.J.K.M.); 2 Department of Microbiology and Immunology, Penn State College of Medicine, 500 University Drive, Hershey, PA 17033, USA

**Keywords:** retroviruses, Rous sarcoma virus, biomolecular condensates, genome selection, dimerization

## Abstract

Retroviral genome selection and virion assembly remain promising targets for novel therapeutic intervention. Recent studies have demonstrated that the Gag proteins of Rous sarcoma virus (RSV) and human immunodeficiency virus type-1 (HIV-1) undergo nuclear trafficking, colocalize with nascent genomic viral RNA (gRNA) at transcription sites, may interact with host transcription factors, and display biophysical properties characteristic of biomolecular condensates. In the present work, we utilized a controlled in vitro condensate assay and advanced imaging approaches to investigate the effects of interactions between RSV Gag condensates and viral and nonviral RNAs on condensate abundance and organization. We observed that the psi (Ψ) packaging signal and the dimerization initiation sequence (DIS) had stabilizing effects on RSV Gag condensates, while RNAs lacking these features promoted or antagonized condensation, depending on local protein concentration and condensate architecture. An RNA containing Ψ, DIS, and the dimerization linkage structure (DLS) that is capable of stable dimer formation was observed to act as a bridge between RSV Gag condensates. These observations suggest additional, condensate-related roles for Gag-Ψ binding, gRNA dimerization, and Gag dimerization/multimerization in gRNA selection and packaging, representing a significant step forward in our understanding of how these interactions collectively facilitate efficient genome packaging.

## 1. Introduction

Retroviruses are single-stranded, positive-sense RNA viruses that cause immunodeficiency syndromes and cancers in animal and human hosts. Much effort has been expended to elucidate the mechanisms underlying retroviral replication, resulting in combination antiretroviral therapies that effectively control infection. However, the high mutation rate of retroviruses can lead to mutations that reduce or abolish the efficacy of current therapies, necessitating the search for novel therapeutic approaches. Some steps in the replication cycle have not yet been effectively exploited for the development of therapeutics, including genomic RNA (gRNA) selection and virion assembly. Therefore, further elucidation of these processes is likely to identify promising targets for therapeutic intervention. This work focuses on understanding the biophysical mechanisms governing recognition of gRNA by retroviral Gag proteins using an innovative in vitro approach that takes into account the multimeric state of Gag rather than studying Gag-gRNA binding in solution.

We recently demonstrated that the Gag proteins of Rous sarcoma virus (RSV) and human immunodeficiency virus type-1 (HIV-1) multimerize and undergo phase separation to form biomolecular condensates (BMCs), membraneless organelles that arise through extensive multivalent interactions between proteins and nucleic acids [1,2,3]. These interactions work in concert to drive phase transitions, separating the constituents from the surrounding cellular milieu and concentrating them within BMCs. Compartmentalization of specific proteins and nucleic acids in this way facilitates various cellular processes throughout the cell. Examples of cellular BMCs include nuclear speckles, paraspeckles, Cajal bodies, nucleoli, transcription complexes, cytoplasmic stress granules, and P bodies [4,5,6,7,8,9,10,11,12,13].

Many viruses have been shown to form replication compartments possessing properties of BMCs, either by co-opting host proteins or encoding viral proteins that form condensates [14,15,16,17,18,19,20,21,22,23]. Recently, we demonstrated that RSV Gag forms phase-contrasted assemblies in vitro and in cells, and that these structures display properties of condensates in the nucleus, cytoplasm, and at the plasma membrane [2]. Phase separation of RSV Gag is driven by two intrinsically disordered regions (IDRs)—conformationally promiscuous regions that are capable of binding multiple partners—within the matrix-p2-p10 (MAp2p10) and nucleocapsid (NC) regions of Gag [2,24,25,26,27]. Like many viral and cellular proteins that form BMCs [28,29], RSV Gag is a nucleic acid binding protein, specifically recognizing the Ψ packaging signal at the 5′ end of the gRNA through its NC domain and packaging a gRNA dimer into each nascent virion [30,31,32,33]. Dimerization of gRNA is facilitated by two cis-acting elements: the dimerization initiation sequence (DIS), which forms a weak dimer via a kissing-loop interaction; and the dimerization linkage structure (DLS), which noncovalently stabilizes the dimer [30,33,34,35,36]. A minimal Ψ-containing sequence necessary for selection by Gag (μΨ [37]) has been identified, and the NC-μΨ interaction has been characterized biochemically and structurally by NMR [38,39]. Gag is also capable of nonspecific interactions—likely through multiple modes including electrostatic, cation–π, and π-π—with other RNAs, as suggested by salt-titration assays that revealed a role of the positively charged matrix (MA) domain in discrimination between Ψ and non-Ψ RNAs by the NC domain [40,41]. Further support for this possibility is the observation that, in the absence of Ψ-containing viral RNA, Gag proteins from multiple retroviruses package a conserved selection of cellular RNAs such as U6 and 7SL [42,43,44]. Interestingly, the association of Gag with nucleic acids has been shown to trigger Gag multimerization, a function that is critical for virion assembly and viral infectivity [45,46,47]. As mentioned above, the RSV Gag-Ψ interaction has been investigated using recombinant Gag in solution, but no study has thus far characterized this interaction in the context of BMCs.

In the present work, we probed the interactions between RSV Gag condensates and a variety of viral and nonviral RNAs using an in vitro system to determine their effect(s) on various biophysical properties of Gag condensates under multiple protein–RNA concentration regimes. Specific emphasis was placed on characterizing the effect(s) of viral RNAs containing the Ψ packaging signal and the dimerization initiation sequence (DIS), as both features are critically important for gRNA packaging [32,48,49,50]. These elements are also known sites of Gag-RNA and RNA-RNA contact and are therefore important for increasing the valency of Gag-RNA condensates. Using purified protein condensation assays and quantitative advanced imaging approaches, we observed significant differences between the effect(s) of viral RNAs containing Ψ and/or the DIS on RSV Gag condensates compared to viral and nonviral RNAs lacking these features. These observations represent a significant step forward in our understanding of how Gag-Ψ, gRNA-gRNA, and Gag-Gag interactions function together to mediate gRNA selection and packaging.

## 2. Materials and Methods

### 2.1. Plasmids

The construct encoding the RSV Gag protein used for purification [pET28(-His) Gag.ΔPR (henceforth referred to as RSV Gag)] was described in [51]. Plasmids for production of viral RNA were generated by cloning the sequences encoding gRNA 156–238 Ψ (also known as μΨ [37]), 156–315 Ψ DIS (also known as MΨ [52,53]), 1248–1409, 1–845 Ψ DIS [36,54], and 1–219/296–845 Ψ ΔDIS [36,54] into a pGEM vector backbone 19 bases (GCGAAUUGGGCCCGACGUC) downstream of the T7 promoter. Templates encoding portions of the hygromycin resistance gene [55], hygromycin-83 and hygromycin-162 RNAs, were generated by PCR corresponding to the indicated lengths (i.e., 83 or 162 bases), starting at the first base. The same forward primer (5′ ATCG**TAATACGACTCACTATA**GATGAAAAAGCCTGAACTCACC 3′) containing the T7 sequence (bolded above) was used alongside unique reverse primers (hygromycin-83: 5′ AGCTGCATCAGGTCGGAGACGC 3′; hygromycin-162: 5′ GGCGCAGCTATTTACCCGCAG 3′) to generate PCR products for use as template for in vitro transcription reactions.

### 2.2. Protein Expression, Purification, and Labeling

Expression and purification of recombinant RSV Gag was performed, as described previously [2,56]. Gag protein was labeled using an Alexa Fluor 488 Microscale Protein Labeling Kit (Thermo Fisher Scientific, Waltham, MA, USA, #A30006) according to the manufacturer’s protocol, and was quantified via NanoDrop One spectrophotometer (Thermo Fisher Scientific, Waltham, MA, USA). The protein was aliquoted into working volumes, flash-frozen in dry ice/ethanol, and stored at −80 °C until use.

### 2.3. RNA Production by In Vitro Transcription and Fluorescent Labeling

The pGEM plasmids referenced above encoding viral RNAs were linearized via restriction enzyme digest followed by gel purification. Linearized plasmids (viral RNAs) or PCR products (hygromycin constructs) were used as template to generate RNAs using a MAXIscript T7 Transcription Kit (Thermo Fisher Scientific, Waltham, MA, USA, #AM1312) according to the manufacturer’s protocol. In order to fluorescently label RNAs, aminoallyl-UTP was included in the reaction at a ratio of 4:1 to unmodified UTP. RNA was subsequently cleaned up via an RNA Clean and Concentrator Kit (Zymo Research Corp., Irvine, CA, USA, #R1014), and length and purity were assessed via agarose gel electrophoresis. RNAs were then labeled using an Alexa Fluor 555 or 647 Reactive Dye Decapack (Invitrogen, Waltham, MA, USA, #A32756 or #A32757, respectively) per the manufacturer’s instructions, followed by quantification using a NanoDrop One spectrophotometer (Thermo Fisher Scientific, Waltham, MA, USA). RNA was aliquoted into working volumes, flash-frozen in dry ice/ethanol, and stored at −80 °C until use. Yeast tRNA (Invitrogen, Waltham, MA, USA, #AM7119) consists of a mixed population of tRNAs ranging between 76 and 90 bases (average 83 bases), and was handled/stored per the manufacturer’s instructions. Polyuridine RNA (20U) was synthesized by Integrated DNA Technologies (IDT, Coralville, IA, USA) and was reconstituted from a lyophilized state in nuclease-free water, aliquoted, and frozen at −80 °C. These last two RNAs lack the modified uridine residues required for fluorescent labeling and were therefore used in their unlabeled state.

### 2.4. RNA Sequences

See Appendix A.

### 2.5. In Vitro Condensate Formation and Imaging

The following buffers were used to form condensates in vitro: (i) No Salt Buffer: 0 mM NaCl, 50 mM HEPES pH 7.5, 1 mM DTT, 1 mM PMSF; (ii) High Salt Buffer: 4000 mM NaCl, 50 mM HEPES pH 7.5, 1 mM DTT, 1 mM PMSF; (iii) Crowding Buffer: 50% *w*/*v* Ficoll-400 in No Salt Buffer. Crowding Buffer was warmed in a 42 °C water bath for at least 1 h to ensure Ficoll-400 was dissolved, with periodic mixing to ensure homogeneity. In reaction tubes, No Salt and High Salt buffers were combined to achieve the desired final NaCl concentration, taking into account the contributions of the components not yet added (protein, RNA, and Crowding Buffer).

Alexa Fluor 488-labeled and unlabeled RSV Gag were thawed to room temperature, centrifuged at 14,000 rpm for 2 min to pellet insoluble material, and transferred to fresh tubes. RSV Gag protein was added to the reaction tubes at a labeled to unlabeled ratio of 1:10. RNA was then added, and the reaction was mixed via gentle pipetting. Crowding Buffer was added to a final concentration of 10% *w*/*v*, the reaction was thoroughly mixed, 6 μL of the reaction was deposited on a coverslip, and a glass slide was placed on top. After 5 min, the coverslip was sealed with nail polish.

Slides were then imaged using a Leica AOBS SP8 FALCON confocal microscope with a 63x/1.2 water objective (Leica Microsystems, Wetzlar, Germany). White light laser (WLL) line excitations of 488 and 647 nm were used in conjunction with hybrid detectors to detect 488-labeled protein and 647-labeled RNA, respectively. A minimum of ten images were acquired at 3x zoom per condition, per experiment, for area/intensity and colocalization analyses. A minimum of three z-stacks were also acquired at 3x and 7x zoom per condition, per experiment, for signal profile and three-dimensional analyses.

### 2.6. Quantitative Image Analysis of In Vitro Condensates

Analysis of condensate area and fluorescence intensity was performed using FIJI (ImageJ v1.54h, National Institutes of Health, Bethesda, MD, USA), as follows. First, images were loaded into FIJI and were duplicated. The duplicate image was then Gaussian-blurred (50 px), and this blurred image was subtracted from the original image to remove background signal. The resulting image was then median-filtered (2 px) to remove single pixel noise and the Triangle auto threshold algorithm was applied. The thresholded area (% of field containing signal) was quantified using the measure function, and this value was recorded. A selection was then created using this thresholded area, and was subsequently applied to the original, unprocessed image. Fluorescence intensity within the selection was quantified, as was the total fluorescence intensity of the image after removing the selection. Fluorescence intensity within the selection was divided by that of the total image, resulting in the proportion of fluorescence intensity within condensates (i.e., the amount phase separated/partitioned into condensates). This workflow was inspired by approaches used by others in the condensate field [57,58,59].

These values were divided by the geometric mean of either the area or intensity values, as appropriate, for RSV Gag in the absence of RNA to determine fold changes in the presence of RNA. These values were then Log_2_-transformed, resulting in positive values for increases and negative values for decreases. Values were plotted as mean ± S.E.M. for each individual RNA copy number (0.5/1/2/4 RNA:3000 Gag). The average of these values at each of the four RNA:Gag ratios, representing the overall effect of each RNA on these two parameters (referred to as “Overall” in this manuscript), were calculated using the following equation:

$$\mathrm{Overall}=\frac{\left(0.5:3000\right)+\left(1:3000\right)+\left(2:3000\right)+\left(4:3000\right)}{4}$$

These values are also plotted as mean ± S.E.M.

To better represent the synergistic effects of changes in area and fluorescence intensity, the Overall Log_2_-transformed fold change values for area and intensity for each RNA were summed, generating a value we dub “Composite Factor” that serves as a general indicator of the amplitude of the effects on these characteristics:

Composite Factor=(Overall Area)+(Overall Intensity)

Signal-based colocalization analysis of deconvolved (Huygens v24.10, Scientific Volume Imaging, Hilversum, Netherlands) images was performed in Imaris Image Analysis Software v10.2.0 (Oxford Instruments, Abingdon, UK). Manders’ Overlap Coefficients were generated and plotted as mean ± S.E.M. A minimum of 9 fields per RNA/copy number were quantified and analyzed.

Fluorescent signal profile analysis was performed on deconvolved images in Imaris Image Analysis Software v10.2.0 (Oxford Instruments, Abingdon, UK) on condensates selected from 3x zoom z-stacks. The Imaris Spot function was used in conjunction with the integrated Intensity Profile MATLAB plugin to measure the fluorescence intensity of Gag and RNA signals across condensates. Clipping planes were selected and spots placed such that the line generated by the MATLAB plugin passed through the center of both Gag and RNA condensate signals. To allow for averaging of condensates of varying sizes and absolute intensities, condensate diameters and intensities were normalized using a protocol inspired by that found in [60]. The maximum Gag fluorescence was set to a value of 100 on the *y*-axis and was placed at position x = 0; the edges of the Gag condensate (i.e., where Gag fluorescence is 0) were set to an x/radius (x/R) value of −1 or 1, and all remaining values were normalized to these. Maximum RNA fluorescence was likewise set to a value of 100 on the *y*-axis and x values remained normalized to Gag, allowing for easy visualization of spatial differences in fluorescence peaks. A minimum of five condensates per condition were assessed in this way.

To make 3D reconstructions, deconvolved images were analyzed in Imaris Image Analysis Software v10.2.0 (Oxford Instruments, Abingdon, UK) using 7x zoom z-stacks. Surface renderings were made from the Gag and RNA signals, and a surface image was taken. The orthogonal clipping plane tool was used to view the internal structure of the condensates, and a clipping image was taken to demonstrate the spatial arrangement of Gag and RNA in condensates.

### 2.7. Förster Resonance Energy Transfer (FRET)

Condensates were formed in vitro, as described above, with the following modifications. The crowding agent used was polyethylene glycol-8000 (PEG-8000) instead of Ficoll-400 and condensate reactions were incubated for 1 h in microcentrifuge tubes at room temperature before mounting on slides. These conditions were more permissive of condensate fusion, resulting in larger condensates more suitable for photobleaching approaches. Alexa Fluor 555 was used in place of Alexa Fluor 647, as it forms a FRET pair with the Alexa Fluor 488-labeled RSV Gag protein. Condensates were analyzed using the FRET AB (Acceptor Bleaching) module on a Leica AOBS SP8 confocal microscope with a 63x/1.2 water objective. FRET efficiency was assessed using the built-in software functionality of Leica Application Suite X v3.5 (LASX, Leica Microsystems, Wetzlar, Germany).

### 2.8. Fluorescence Recovery After Photobleaching (FRAP)

Condensates were formed in the same manner as for FRET experiments above (PEG-8000, 1 h incubation prior to plating) except that RNA was labeled with Alexa Fluor 647. Condensates were imaged using the FRAP module on a Leica AOBS SP8 confocal microscope with a 63x/1.2 water objective (Leica Microsystems, Wetzlar, Germany). For each image, three regions of interest (ROIs) were utilized: (1) an ROI placed at the center of the condensate, which was bleached; (2) an ROI of a separate condensate within the same field, to account for bleaching of areas outside ROI 1; and (3) an ROI away from condensates, to allow for background subtraction. Five pre-bleach images (1 image/second) were taken with a WLL line excitation of 488 at 5% laser power, followed by ten bleaching frames (1 image/second) utilizing 100% laser power. Recovery images were then collected every minute for five minutes using the 488-laser line at 5% laser power. ROIs that were bleached were done so to ensure a bleaching depth of ≤60% of the starting fluorescence intensity. To calculate the mobile fractions and t_1/2_ values for each condition, the web application easyFRAP-web (https://easyfrap.vmnet.upatras.gr/, accessed on 15 October 2024) was used [61].

### 2.9. Quantitative Image and Statistical Analyses

Image analysis was performed in Leica Application Suite X v3.5 (LASX, Leica Microsystems, Wetzlar, Germany), Imaris Image Analysis Software v10.2.0 (Oxford Instruments, Abingdon, UK), and/or FIJI (ImageJ v1.54h, National Institutes of Health, Bethesda, MD, USA), as indicated. Huygens Deconvolution software v24.10 (Scientific Volume Imaging, Hilversum, The Netherlands) was utilized for deconvolution. Data were analyzed and plotted using Microsoft Excel (Microsoft, Redmond, WA, USA) and GraphPad Prism v10.2.2 (GraphPad Software, Boston, MA, USA).

### 2.10. RNA Secondary Structure Prediction

Secondary structures of the RNAs used in this manuscript were predicted using the RNAstructure web server (https://rna.urmc.rochester.edu/RNAstructureWeb/index.html, accessed 19 November 2024; Mathews Lab, University of Rochester [62,63]) and were modeled using RNACanvas (https://rnacanvas.app/, accessed 21 November 2024) [64]. The partition and MaxExpect algorithms were used in conjunction to generate structures with the highest probability of being correct.

## 3. Results

### 3.1. Elucidating the Effect of Viral and Nonviral RNAs on the Biophysical Properties of RSV Gag Biomolecular Condensates

**Figure 1:**
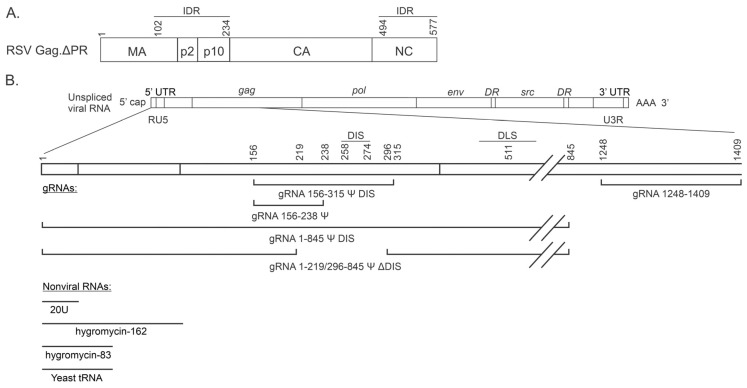
RSV Gag.ΔPR and gRNA constructs. (**A**) RSV Gag is a multidomain protein consisting of matrix (MA), p2, p10, capsid (CA), nucleocapsid (NC), and protease (PR) domains. To prevent autoproteolysis of Gag, the carboxy-terminal PR domain has been removed from this recombinant protein to generate RSV Gag.ΔPR. The NC domain of RSV Gag specifically recognizes and binds to the Ψ packaging sequence in the viral genomic RNA (gRNA); both NC and the positively charged MA domain are capable of nonspecific interactions with nucleic acids. This protein also contains two intrinsically disordered regions (IDRs; amino acids ~102–234 and ~494–577) that contribute to biomolecular condensation. (**B**) Schematic of RNA constructs used in this manuscript. Regions encoding viral gRNAs are indicated in relation to the unspliced viral RNA from which they are derived. Nonviral RNAs are depicted below; Ψ, psi packaging signal; DIS, dimerization initiation sequence; DLS, dimerization linkage structure.

RSV Gag is a 701 amino acid, multidomain protein that is responsible for viral genome selection and orchestrates virion assembly within cells (Figure 1A) [32]. Selection of gRNA is mediated by the NC domain, which specifically recognizes the Ψ packaging signal in the 5′ leader sequence of gRNA. NC also binds nucleic acids nonspecifically via electrostatic mechanisms mediated by positively charged amino acids surrounding its two Zinc finger motifs [32]. The RSV MA domain contains basic residues that form a positively charged interface that binds negatively charged phospholipids such as phosphatidylinositol-(4,5)-bisphosphate [PI(4,5)P_2_] in the plasma membrane, and likely contributes to nonspecific, electrostatic binding to additional cellular nucleic acids [42,43,44,65,66,67,68]. Regions of the MA, NC, p2, and p10 domains are intrinsically disordered, allowing them to adopt multiple conformations and interact with variable protein partners, promoting self-assembly of RSV Gag into BMCs in vitro and in cells [2]. However, it is not known how RNA affects the biophysical properties of RSV Gag BMCs. Given that RSV Gag encounters both spliced and unspliced viral RNAs (vRNAs; both of which contain Ψ) in the nucleus, as well as cellular RNAs, it is not well understood how Gag preferentially selects unspliced vRNA for packaging. One possibility is that gRNA dimerization occurs co-transcriptionally, in a similar manner to murine leukemia virus (MLV) [50,69], and that the dimerized gRNA is preferentially selected for packaging. In support of this idea, we previously demonstrated that RSV gRNA forms homodimers in the nucleus that are preferentially packaged [50]. It remains uncertain whether the RSV Gag-gRNA association occurs before or after gRNA dimerization, but there is evidence for the association of Gag and nascent gRNA in transcriptional condensates [2,24,70], supporting the possibility that co-transcriptional dimerization and gRNA selection are intimately linked.

To probe these important questions, we compared several gRNA sequences derived from the Prague C strain of RSV [71] for their ability to influence properties of Gag condensates in vitro (Figure 1B). We tested the minimum Ψ-containing RNA sequence necessary for selection by Gag, gRNA 156–238 Ψ (μΨ [37]), and a longer version of this Ψ-containing RNA that also contains the DIS, gRNA 156–315 Ψ DIS (MΨ [52,53]). To assess the effect(s) of gRNA dimerization on Gag condensate formation, we utilized two longer Ψ-containing gRNAs that were relatively similar but either contained or lacked the DIS: gRNA 1–845 Ψ DIS and gRNA 1–219/296–845 Ψ ΔDIS (referred to as mutant 15–4 or Bal31 in prior publications, which is defective in viral replication assays) [36,48,54]. The last gRNA sequence used was a 161 nucleotide (nt)-long region of gRNA spanning nt 1248–1409 (gRNA 1248–1409) in the capsid (CA) coding region, a sequence that is not known to interact specifically with Gag.

Comparisons were made between gRNA sequences of similar lengths, and were extended to include nonviral RNAs as well. RNAs derived from the hygromycin resistance gene [55], hygromycin-83 and hygromycin-162, were utilized, as was a commercially available preparation consisting of a mixed population of tRNAs. Finally, 20U was included as a short, unstructured polyanion of simple composition, representative of the type used in many published condensate studies [72,73,74].

To investigate the influence of RNA length on BMC formation, we divided the data into three groups: “short” RNAs, ≤83 bases (gRNA 156–238 Ψ, hygromycin-83, yeast tRNA, 20U); “medium” RNAs, 159–162 bases (gRNA 156–315 Ψ DIS, gRNA 1248–1409, hygromycin-162); and “long” RNAs, 767–845 bases (gRNA 1–845 Ψ DIS, gRNA 1–219/296–845 Ψ ΔDIS).

### 3.2. Viral and Nonviral RNAs Greatly Influence RSV Gag Condensates at a Low Gag Concentration (2.5 μM) and at a Ratio of 2 RNAs per 3000 Gag Molecules

**Figure 2:**
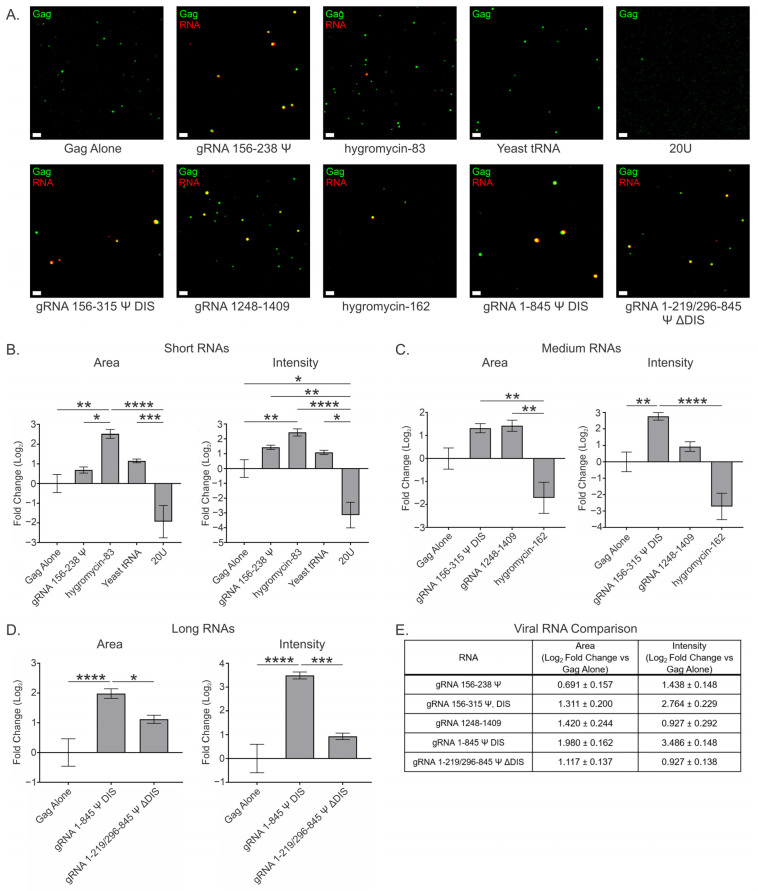
Effect of RNA on 2.5 μM RSV Gag condensates, 2 RNA:3000 Gag. (**A**) Representative images of RSV Gag (green)-RNA (red) condensates; scale bars = 2 μm. (**B**–**D**) Fold changes (Log_2_) in condensate area and intensity (left and right panels, respectively) for each of the three RNA length groups ((**B**), short; (**C**), medium; (**D**), long) are displayed as mean values ± S.E.M. (n ≥ 9). Statistical significance was determined by Kruskal–Wallis test with Dunn’s post-hoc test (****, *p* ≤ 0.0001; ***, *p* ≤ 0.001; **, *p* ≤ 0.01; *, *p* ≤ 0.05). (**E**) Table summarizing values from (**B**–**D)** for viral RNAs.

It is well documented that RNAs have a modulatory effect on protein condensates, in many cases serving as a scaffold and promoting condensation at protein concentrations where few condensates would otherwise form (reviewed in [75]). We previously found that few RSV Gag condensates formed at concentrations below 5 μM in the absence of RNA, and that the presence of RNA was able to promote condensation of HIV Gag at 2.5 μM [2,3]. To determine the influence of RNA on RSV Gag BMC formation at 2.5 μM, we performed in vitro condensate assays in the presence of viral or nonviral RNAs. We adopted a population-level approach and assessed the fold change in total microscope field coverage (area) and the proportion of total Gag fluorescence intensity within that area (intensity) as indicators of the amount of Gag condensation in the presence of various RNAs (Figure 2). This approach has several advantages over assessing condensate number and size; most notably, it accounts for instances when the relationship between condensate size and the amount of protein within condensates is not linear. Similar approaches have been effectively utilized by other groups to study condensate formation in vitro [57,58,59].

It is well known that RSV and other retroviruses package two copies of gRNA as a noncovalently bound dimer in virions, although studies indicate that there is some variability in the number of copies of Gag per virion [76,77]. Best estimates place the number of copies of Gag somewhere between 1500 and 5000, depending on the specific retrovirus. Notably, the number of Gag molecules within virions was found in one study to greatly exceed the number necessary to form the mature viral core [77].

To account for this variability in the ratio of Gag to RNA, we chose to assess multiple ratios of RNA to Gag (0.5, 1, 2, and 4 copies of RNA per 3000 copies of Gag), to determine whether having fewer or more copies of RNA than two per virion would alter the properties of Gag condensates (Figure 2 and Figure S1). The data for two copies of RNA per 3000 copies of Gag are displayed in the main text, but the data for the additional ratios of RNA to Gag are presented as Supplemental Data.

At two copies of RNA:3000 Gag molecules, all short RNAs except 20U resulted in an increase in condensate area and intensity at 2.5 μM Gag to different degrees (Figure 2B and Table 1). The shortest viral RNA, gRNA 156–238 Ψ, affected condensate area the least of any RNA (0.691 ± 0.157), although it had about twice the effect on condensate intensity (1.438 ± 0.148) as it did on area, suggesting the Gag-Ψ interaction creates an intra-condensate environment permissive to the inclusion of additional Gag molecules, resulting in densely packed condensates. In contrast, the nonviral hygromycin-83 RNA increased both condensate area (2.515 ± 0.224) and intensity (2.432 ± 0.239) by similar amounts, suggesting that this RNA is serving as a scaffold. Hygromycin-83 was the only short RNA to exhibit a significant increase in either area or intensity when compared to Gag alone. The increase in area with hygromycin-83 was also significant when compared to all other short RNAs apart from yeast tRNA. Yeast tRNA exhibited smaller increases in area (1.150 ± 0.095) and intensity (1.092 ± 0.145) than hygromycin-83, but maintained this linear relationship between parameters, suggesting a similar mechanism as hygromycin-83. Finally, 20U RNA decreased both condensate area (−1.937 ± 0.821) and intensity (−3.146 ± 0.865) in a manner consistent with resolubilization of Gag. One possibility is that the small, unstructured nature of 20U allows it to deeply infiltrate condensates, disrupting Gag-Gag interactions throughout. The decrease in area observed with this RNA was significant when compared to both hygromycin-83 and yeast tRNA, but was not significant when compared to Gag alone. The decrease in intensity, however, was significant compared to Gag alone and all other short RNAs.

The medium-length viral RNAs—gRNA 156–315 Ψ DIS and gRNA 1248–1409—increased condensate area and intensity, whereas the nonviral hygromycin-162 decreased both of these parameters at 2.5 μM Gag (Figure 2C and Table 1). Similar to the shorter Ψ-containing RNA, gRNA 156–315 Ψ DIS affected condensate intensity (2.764 ± 0.229) about twice as much as condensate area (1.311 ± 0.200), suggesting a similarly permissive environment and densely packed architecture. This increase in condensate intensity was found to be significant when compared to both Gag alone and hygromycin-162. In contrast, gRNA 1248–1409, which does not contain Ψ, exhibited an area increase (1.420 ± 0.244) ~1.5 times larger than the increase in intensity (0.927 ± 0.292). This observation suggests that gRNA 1248–1409 serves as a scaffold similar to the nonviral RNAs, but results in condensates with even less densely packed Gag. One possibility for this observation is that such regions within the RSV gRNA may be crucial for the prevention of pathologically dense Gag condensates that lose intra-condensate mobility or result in irreversible aggregates, as described for cellular RNA binding proteins (i.e., TDP-43 and hnRNPA1) [72,78,79,80,81]. The presence of hygromycin-162 resulted in a less dramatic resolubilization of Gag as seen with 20U, exhibiting decreases in both BMC area (−1.712 ± 0.678) and intensity (−2.724 ± 0.805). No significant difference was observed when comparing the effects of the medium RNAs on area to Gag alone, but both viral RNAs did exhibit significantly higher area values when compared to hygromycin-162.

Of the two long RNAs examined, only gRNA 1–845 Ψ DIS displayed both area (1.980 ± 0.162) and intensity (3.486 ± 0.148) increases that were significant when compared to Gag alone (Figure 2D and Table 1). Interestingly, this RNA appeared to function similarly to the short- and medium-length Ψ-containing RNAs in terms of densely packed condensate architecture. These values were also significant when compared to the area (1.117 ± 0.137) and intensity (0.927 ± 0.138) increases seen with gRNA 1–219/296–845 Ψ ΔDIS. This finding is interesting considering that the main difference between these two RNAs is the presence of the DIS, suggesting that dimerization of gRNA 1–845 Ψ DIS may account for the significant differences observed. In support of this possibility, all Ψ-containing RNAs, with the exception of gRNA 1–219/296–845 Ψ ΔDIS, displayed area and intensity increases that increased in order of RNA length (gRNA 1–845 Ψ DIS > gRNA 156–315 Ψ DIS > gRNA 156–238 Ψ) (Figure 2B–E and Table 1), but the increases observed between gRNA 156–315 Ψ DIS and gRNA 1–845 Ψ DIS were not proportional to their length. The exclusion of gRNA 1–219/296–845 Ψ ΔDIS from this pattern may also be caused by the perturbation of secondary structure compared to gRNA 1–845 Ψ DIS, which is possible given that their predicted secondary structures do differ somewhat in the region surrounding the deletion (Figure S8). Taken together, these observations suggest that Ψ and the DIS function synergistically to concentrate Gag and gRNA within condensates, thereby facilitating efficient retroviral genome selection.

### 3.3. Overall Effect of Viral and Nonviral RNAs at a Low Gag Concentration (2.5 μM) and Range of RNA Ratios (0.5, 1, 2, and 4) per 3000 Gag Molecules

**Figure 3:**
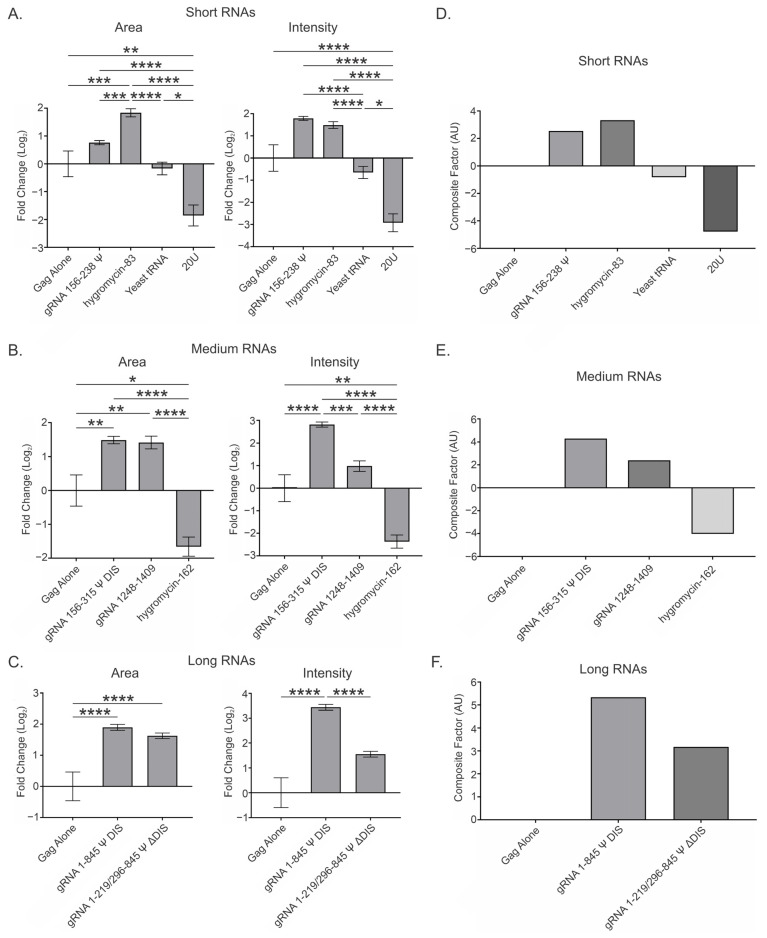
Overall effect of RNA on 2.5 μM RSV Gag condensates. (**A**–**C**) Fold changes (Log_2_) in condensate area and intensity (left and right panels, respectively) for each of the three RNA length groups ((**A**), short; (**B**), medium; (**C**), long) are displayed as mean values ± S.E.M. (n ≥ 39). Statistical significance was determined by Kruskal–Wallis test with Dunn’s post-hoc test (****, *p* ≤ 0.0001; ***, *p* ≤ 0.001; **, *p* ≤ 0.01; *, *p* ≤ 0.05). Composite Factor values, representing the overall effect of each RNA across four RNA copy number conditions (0.5, 1, 2, 4 RNA:3000 Gag), are displayed (**D**–**F**).

Next, we took the mean of the data from all four RNA:Gag ratios to generate a value (referred to as “Overall”) that was reflective of the likely heterogeneous ratios of RNA:Gag present in transcriptional condensates (Figure 3A–C and Table 2). These mean values allowed us to make comparisons among the different RNAs tested and to summarize whether certain RNAs were more likely to promote or antagonize Gag condensation. The Overall values for the effect of the RNAs on area and intensity were used to generate an additional parameter (“Composite Factor”, equal to the sum of the Overall area and intensity values), which represents the relative magnitude of the effect of any given RNA (Figure 3D–F). These Overall and Composite Factor values should be interpreted alongside the individual values for area and intensity found in the main text and Supplemental Data (Figure 2 and Figure S1, and Tables S1–S3), and provide a useful summary of how each RNA affects these condensate parameters.

The Overall values show variable effects on the area and intensity of Gag condensates in the presence of each of the short RNAs (Figure 3A and Table 2). Viral gRNA 156–238 Ψ affected condensate area (0.762 ± 0.075) the least of any of the short RNAs, and this value was similar to that seen in the 2 RNA:3000 Gag data (0.691 ± 0.157), suggesting relative consistency in area values between datasets. Each of the additional RNA:Gag ratios tested for this construct exhibited marginally larger increases in intensity, resulting in a greater Overall intensity value (1.785 ± 0.090) and therefore a greater difference (~12%) between the effect of this RNA on area and intensity. Therefore, the densely packed condensate environment promoted by gRNA 156–238 Ψ appears to be independent of RNA:Gag ratio. For hygromycin-83, incorporation of the additional datasets decreased both area (1.833 ± 0.147) and intensity (1.488 ± 0.150) values greatly. Notably, these changes resulted in a ratio between area and intensity values that suggests a more prominent role in spacing out Gag within condensates. Despite the decrease in area, hygromycin-83 was still the only short RNA to significantly increase area compared to Gag alone, and this increase was also significant in relation to all other short RNAs. In contrast, the decrease in condensate intensity was not significant when compared to Gag alone. The largest difference associated with the inclusion of these additional RNA:Gag ratios was observed for yeast tRNA, as all datasets exhibited values for these parameters quite disparate from that observed with 2 RNA:3000 Gag. These differences resulted in Overall values that were of lower magnitude and were negative for area (−0.167 ± 0.228) and intensity (−0.653 ± 0.273). The yeast tRNA intensity value was found to be significantly less than that of both gRNA 156–238 Ψ and hygromycin-83. These results for yeast tRNA suggest that its effect on Gag condensates was heavily dependent on RNA:Gag ratio. The Overall area (−1.857 ± 0.377) and intensity (−2.921 ± 0.403) values for 20U were similar to those observed with 2 RNA:3000 Gag, suggesting little impact of RNA:Gag ratio on the resolubilization effect of this RNA. However, the negative impact of 20U RNA on both area and intensity was statistically significant compared to Gag alone and the other short RNAs.

Interestingly, the Overall area and intensity values for the medium-length RNAs closely agreed with those of the 2 RNA:3000 Gag dataset (Figure 3B and Table 2). All Overall area and intensity values, except for the hygromycin-162 intensity value (−2.369 ± 0.290), were within 0.200 of that for 2 RNA:3000 Gag (Figure 2C and Figure 3B; Table 1 and Table 2), suggesting that these RNAs exerted similar effects regardless of RNA:Gag ratio. Due to increased statistical power, both viral RNAs—gRNA 156–315 Ψ DIS (1.489 ± 0.107) and gRNA 1248–1409 (1.416 ± 0.187)—exhibited a significant increase in condensate area compared to Gag alone, in addition to retaining their statistical significance versus hygromycin-162 (−1.661 ± 0.281), which was now significantly lower than Gag alone. As for intensity, the value for gRNA 156–315 Ψ DIS (2.813 ± 0.115) was significantly different when compared to Gag alone and both other medium RNAs, while that for gRNA 1248–1409 (0.979 ± 0.230) was significant compared to hygromycin-162 (−2.369 ± 0.290).

Area (1.896 ± 0.095) and intensity (3.439 ± 0.117) values for gRNA 1–845 Ψ DIS were also quite similar to those at 2 RNA:3000 Gag (1.980 ± 0.162 and 3.486 ± 0.148, respectively), but increases (~0.600) were observed in the area (1.625 ± 0.090) and intensity (1.552 ± 0.117) values for gRNA 1–219/296–845 Ψ ΔDIS (Figure 3C and Table 2). These increases brought the area values for these two RNAs into closer agreement, resulting in both being significant compared to Gag alone. However, the increase in intensity for gRNA 1–219/296–845 Ψ ΔDIS did not have a similar effect on altering significance between these RNAs, nor between gRNA 1–219/296–845 Ψ ΔDIS and Gag alone, resulting in the same findings as with 2 RNA:3000 Gag. Again, it is interesting that gRNA 156–238 Ψ, gRNA 156–315 Ψ DIS, and gRNA 1–845 Ψ DIS all appeared relatively unaffected by the RNA:Gag ratio, whereas gRNA 1–219/296–845 Ψ ΔDIS RNA exhibited a slight dependence upon the ratio. Similarly to 2 RNA:3000 Gag, all Ψ-containing RNAs except gRNA 1–219/296–845 Ψ ΔDIS displayed both area and intensity increases that increased in order of RNA length (Figure 3A–C and Table 2). In contrast to that condition, however, the Overall area value for gRNA 1–219/296–845 Ψ ΔDIS was between those of gRNA 156–315 Ψ DIS and gRNA 1–845 Ψ DIS.

When combining these Overall area and intensity values into Composite Factor values, all viral RNAs plus hygromycin-83 exhibited net increases in condensation, ranked as follows: gRNA 1–845 Ψ DIS > gRNA 156–315 Ψ DIS > hygromycin-83 > gRNA 1–219/296–845 Ψ ΔDIS > gRNA 156–238 Ψ > gRNA 1248–1409. In contrast, the remaining nonviral RNAs exhibited net decreases in condensation, ranked as follows: 20U < hygromycin-162 < yeast tRNA (Figure 3D–F and Table S7).

### 3.4. Viral and Nonviral RNAs Modestly Affect Gag Condensates at a High Gag Concentration (10 μM) at a Ratio of 2 RNAs per 3000 Gag Molecules

**Figure 4:**
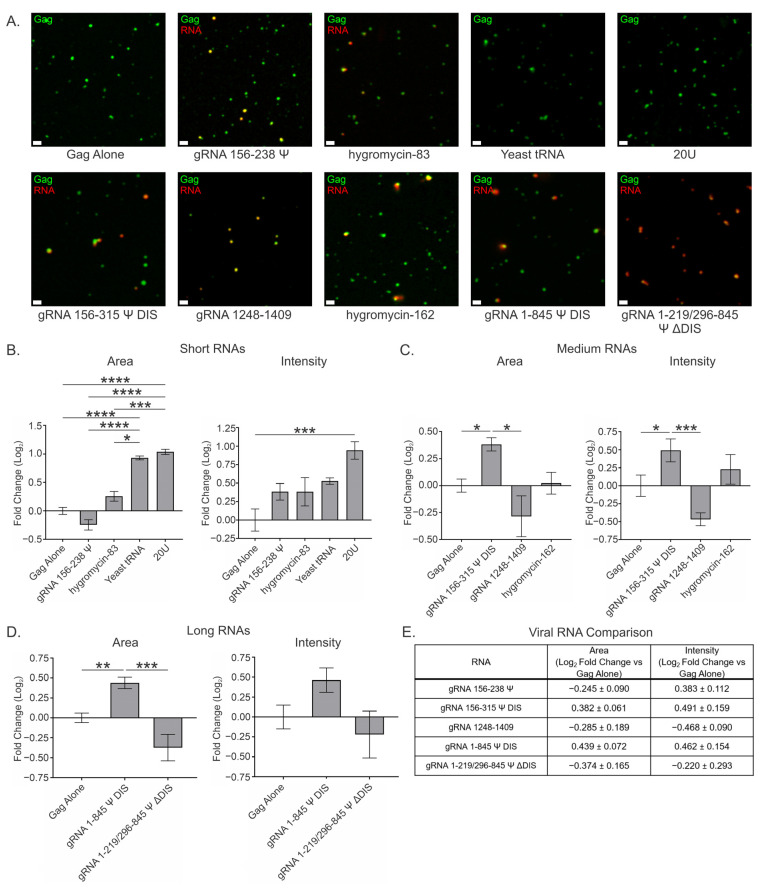
Effect of RNA on 10 μM Gag condensates, 2 (or 1) RNA:3000 Gag. (**A**) Representative images of RSV Gag (green)-RNA (red) condensates; scale bars = 2 μm. (**B**–**D**) Fold changes (Log_2_) in condensate area and intensity (left and right panels, respectively) for each of the three RNA length groups ((**B**), short; (**C**), medium; (**D**), long) are displayed as mean values ± S.E.M. (n ≥ 15). Statistical significance was determined by Kruskal–Wallis test with Dunn’s post-hoc test (****, *p* ≤ 0.0001; ***, *p* ≤ 0.001; **, *p* ≤ 0.01; *, *p* ≤ 0.05). (**E**) Table summarizing values from (**B**–**D**) for viral RNAs.

Because the sensitivity of Gag condensates to the presence of RNA may also differ based on protein concentration, we performed the same experiments using a higher concentration of Gag (10 μM) and asked whether condensates formed under a higher protein concentration regime would respond to the presence of viral and nonviral RNAs differently. We observed that, at the same ratios of RNA:Gag, the effects on condensates formed with 10 μM Gag were far more modest and somewhat more variable (Figure 4 and Figure S2, Table 3).

At 10 μM Gag and 2 RNA:3000 Gag, all short RNAs increased condensate area and intensity, with the exception of gRNA 156–238 Ψ, which increased intensity but decreased area (Figure 4A,B and Table 3). The decrease in area in the presence of gRNA 156–238 Ψ (−0.245 ± 0.090) was moderate and was not statistically significant when compared to Gag alone, nor was the increase in intensity (0.383 ± 0.112). However, it is worth noting that the trend of increasing condensate intensity, while having a much less noticeable effect on condensate area, was still present with gRNA 156–238 Ψ. Of those RNAs that increased condensate area, hygromycin-83 (0.256 ± 0.084) increased area the least of the short RNAs, in stark contrast to what was observed at 2.5 μM where it increased area the most of all the short RNAs tested (Figure 2B and Figure 4A,B). Like gRNA 156–238 Ψ, neither this nor the increase in intensity (0.256 ± 0.084) were significant compared to Gag alone. The relative similarity in area and intensity increases at 2 RNA:3000 Gag was, however, similar to that observed at 2.5 μM Gag.

Surprisingly, the presence of yeast tRNA increased area (0.931 ± 0.035) to a relatively similar degree at both 10 μM and 2.5 μM Gag (Figure 2B and Figure 4A,B), while only increasing intensity (0.528 ± 0.043) about half that amount. As discussed previously, this observation is consistent with less tightly packed Gag proteins within condensates. This relatively large increase in area was significant when compared to Gag alone, gRNA 156–238 Ψ, and hygromycin-83. Once again, 20U RNA exhibited the largest effect on condensate area (1.036 ± 0.045) and intensity (0.943 ± 0.118) of any short RNA tested, although the effect was more modest than at 2.5 μM and was no longer indicative of resolubilization (Figure 2B and Figure 4A,B). The observation that the condensates formed at 10 μM are no longer resolubilized by 20U, and instead benefit from the presence of this RNA, suggests (a) an altered condensate architecture recalcitrant to infiltration by 20U, (b) the neutralization of high positive charge caused by densely packed Gag, or (c) a combination of the two. Like yeast tRNA, the area increase exhibited by 20U was significant when compared to Gag alone, gRNA 156–238 Ψ, and hygromycin-83. In contrast, the increase in intensity was also significant when compared to Gag alone.

The medium-length RNAs also exhibited marked differences compared to 2.5 μM Gag at the two RNA copy number, with both gRNA 156–315 Ψ DIS and hygromycin-162 exhibiting increases in area and intensity, and gRNA 1248–1409 now resulting in decreases for both parameters (Figure 4A,C and Table 3). Viral gRNA 156–315 Ψ DIS exhibited relatively similar increases in area (0.382 ± 0.061) and intensity (0.491 ± 0.159), both of which were found to be significant when compared to Gag alone and gRNA 1248–1409. These relatively modest increases may once again be explained by a decreased sensitivity to the contributions of RNA, likely driven by changes in condensate architecture.

As mentioned above, gRNA 1248–1409 displayed decreases in area (−0.285 ± 0.189) and intensity (−0.468 ± 0.090), although these were not significant when compared to Gag alone. Despite their lack of significance, it is interesting that these values are both negative at 10 μM Gag, supporting the previously mentioned possibility that regions of gRNA presumably not involved in the Gag-Ψ interaction may serve to balance or moderate the forces driving condensation. Finally, hygromycin-162 displayed the smallest effects on area (0.022 ± 0.101) and intensity (0.229 ± 0.206) of any medium RNA tested (Figure 4A,C and Table 3). Like 20U, both of these changes were positive, in contrast to the relatively large decreases in these parameters observed at 2.5 μM Gag (Figure 2B), suggesting a similar mechanism is at play with these two RNAs.

Due to limitations of RNA concentration, the long RNAs were not made at sufficiently high concentration to use at the ratio of 2 RNA:3000 Gag, so they were tested using one copy of RNA per 3000 Gag at 10 μM Gag (Figure 4A,D and Table 3). Despite the difference in RNA copy number, gRNA 1–845 Ψ DIS exhibited very similar effects on area (0.439 ± 0.072) and intensity (0.462 ± 0.154) as the medium, Ψ-containing gRNA 156–315 Ψ DIS RNA (Figure 4A,C and Table 3). These intensity values were also closely aligned with that of the short, Ψ-containing gRNA 156–238 Ψ RNA (Figure 4A,B and Table 3). Taken together, these observations suggest that the contributions of RNA length—and more importantly, the DIS—seen at 2.5 μM are tempered at 10 μM Gag. Interestingly, gRNA 1–219/296–845 Ψ ΔDIS behaved much more similarly to gRNA 1248–1409, exhibiting decreases in both area (−0.374 ± 0.165) and intensity (−0.220 ± 0.293), in contrast to the corresponding values at 2.5 μM Gag (Figure 2C and Figure 4A,D, and Table 3). The area value for gRNA 1–845 Ψ DIS was found to be significant compared to both Gag alone and gRNA 1–219/296–845 Ψ ΔDIS, but there was no significant difference between intensity values at this protein concentration.

An interesting observation from these experiments is that some of the RNAs which antagonized condensation at 2.5 μM Gag promoted condensation at this higher protein regime, and vice-versa. These findings potentially highlight the role of protein concentration in condensate architecture and accessibility by RNAs. Additionally, the length-ordered increases in area and intensity amongst the Ψ-containing viral RNAs (excluding gRNA 1–219/296–845 Ψ ΔDIS) seen at 2.5 μM and 2 RNA:3000 Gag are entirely absent, further highlighting differences in Gag condensate–RNA interaction at 10 μM (Figure 4E).

### 3.5. Overall Effect of Viral and Nonviral RNAs at High Gag Concentration (10 μM) and Range of RNA Ratios (0.5, 1, 2, and 4) per 3000 Gag Molecules

**Figure 5:**
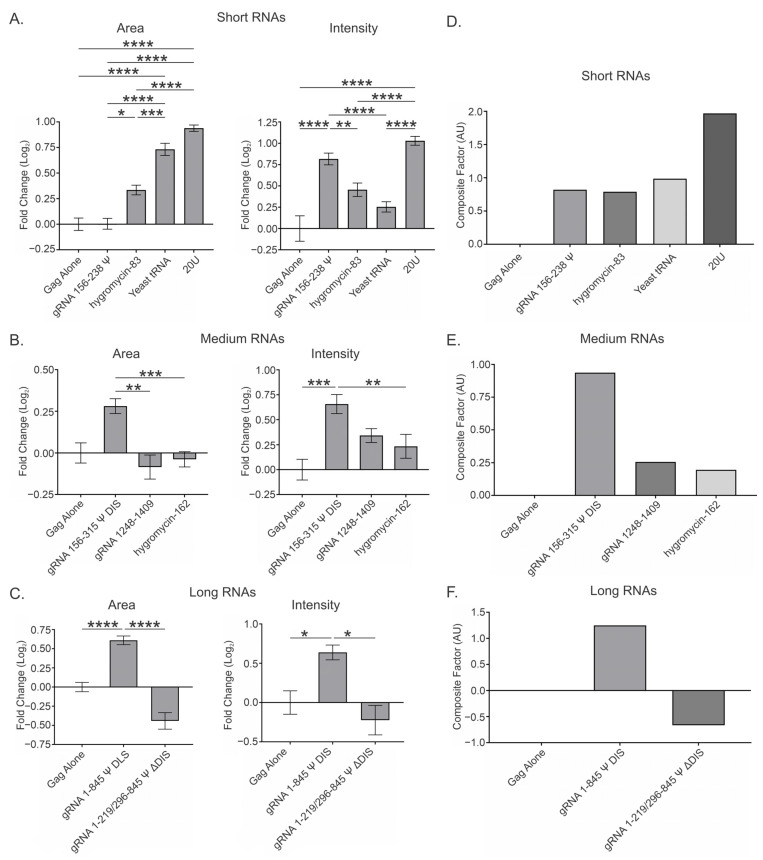
Overall effect of RNA on 10 μM RSV Gag condensates. (**A**–**C**) Fold changes (Log_2_) in condensate area and intensity (left and right panels, respectively) for each of the three RNA length groups ((**A**), short; (**B**), medium; (**C**), long) are displayed as mean values ± S.E.M. (n ≥ 60). Statistical significance was determined by Kruskal–Wallis test with Dunn’s post-hoc test (****, *p* ≤ 0.0001; ***, *p* ≤ 0.001; **, *p* ≤ 0.01; *, *p* ≤ 0.05). Composite Factor values, representing the overall effect of each RNA across four RNA copy number conditions (0.5, 1, 2, 4 RNA:3000 Gag), are displayed (**D**–**F**).

As seen at 2.5 μM Gag, incorporating the additional RNA copy number data (Figure S2 and Tables S4–S6) to generate the Overall values had variable effects on the area and intensity values in the presence of the short RNAs (Figure 5A and Table 4). Notably, gRNA 156–238 Ψ had essentially no effect on condensate area (0.004 ± 0.053) but displayed one of the largest increases in intensity (0.816 ± 0.069) of the short RNAs tested, once again supporting the role of the Gag-Ψ interaction in promoting densely packed condensates. The Overall increases in area (0.334 ± 0.048) and intensity (0.455 ± 0.079) with hygromycin-83 were very similar to those at 2 RNA:3000 Gag, and yeast tRNA induced changes in area (0.732 ± 0.060) and intensity (0.254 ± 0.060) still indicative of less tightly packed Gag molecules within these condensates. The increases in area (0.939 ± 0.031) and intensity (1.029 ± 0.052) exhibited by 20U were largely unaffected by the presence of the additional RNA copy number datasets, supporting the same conclusions as mentioned above.

Similar to the short RNAs, the Overall values for the medium RNAs were variably affected by the presence of the additional datasets (Figure 5B and Table 4). A slight widening of the area (0.281 ± 0.045) and intensity (0.656 ± 0.096) values for gRNA 156–315 Ψ DIS was observed, bringing their ratio more in line with that observed at 2.5 μM Gag (i.e., ~2-fold). This change was largely driven by the 0.5 and 1 RNA:3000 Gag data, suggesting that a lower ratio of RNA:Gag may be optimal at 10 μM Gag (Figure S2, Tables S4 and S5). The modestly decreased area and intensity observed with gRNA 1248–1409 at 2 RNA:3000 Gag were tempered by the inclusion of the other copy numbers, resulting in an area (−0.085 ± 0.072) value much closer to zero and a positive intensity (0.341 ± 0.069) value. These data suggest that gRNA 1248–1409 specifically antagonizes the addition of more Gag to condensates at 2 RNA:3000 Gag, but promotes its addition at these other ratios of RNA:Gag. Finally, there was very little change in the hygromycin-162 area (−0.038 ± 0.046) and intensity (0.233 ± 0.119) values compared to those of 2 RNA:3000 Gag.

The Overall values for gRNA 1–845 Ψ DIS support the same conclusion drawn for gRNA 156–315 Ψ DIS (Figure 5C and Table 4). When incorporating the 0.5 RNA:3000 Gag data, there was a modest increase in both area (0.610 ± 0.057) and intensity (0.638 ± 0.094) values for gRNA 1–845 Ψ DIS, suggesting that a lower ratio of RNA:Gag may be optimal at 10 μM Gag. Combined with relatively unchanged area (−0.442 ± 0.108) and intensity (−0.224 ± 0.187) values for gRNA 1–219/296–845 Ψ ΔDIS, gRNA 1–845 Ψ DIS displayed significantly higher values than Gag alone and gRNA 1–219/296–845 Ψ ΔDIS for both parameters, as opposed to only area in the 1 RNA:3000 Gag dataset.

Composite Factor values for 10 μM Gag condensates were all positive, with the exception of gRNA 1–219/296–845 Ψ ΔDIS, and were ranked as follows: 20U > gRNA 1–845 Ψ DIS > yeast tRNA > gRNA 156–315 Ψ DIS > gRNA 156–238 Ψ > hygromycin-83 > gRNA 1248–1409 > hygromycin-162 > gRNA 1–219/296–845 Ψ ΔDIS (Figure 5D–F and Table S7). As mentioned above, the effect of RNA on Gag condensates was far more modest at 10 μM Gag, exhibiting only ~0.26 times the effect observed at 2.5 μM based on Composite Factors. Additionally, all RNAs with the exception of yeast tRNA displayed a much greater effect on condensates formed using 2.5 μM Gag. This difference in sensitivity to RNA can likely be explained by differences in condensate architecture that both restrict access to the condensate interior and buffer nonspecific interactions. This assertion is supported by a few observations, most notably that 20U displayed the greatest Composite Factor at 10 μM Gag (1.968), suggesting that its small size allows it better access to regions not accessible by the larger RNAs. Next, the Ψ-containing gRNA 156–238 Ψ, gRNA 156–315 Ψ DIS, and gRNA 1–845 Ψ DIS RNAs exhibit relatively similar Composite Factors (0.82, 0.93, and 1.25, respectively) but very different intensity–area ratios (204:1, 2.33:1, and 1.05:1, respectively), suggesting disparate mechanisms for their observed effects, likely driven by a size-related exclusion of the larger RNAs. Finally, the gRNA 156–238 Ψ Composite Factor is affected the least by Gag concentration (Table S7).

### 3.6. RSV Gag Colocalization at 2.5 μM and 10 μM with Viral and Nonviral RNAs

**Figure 6:**
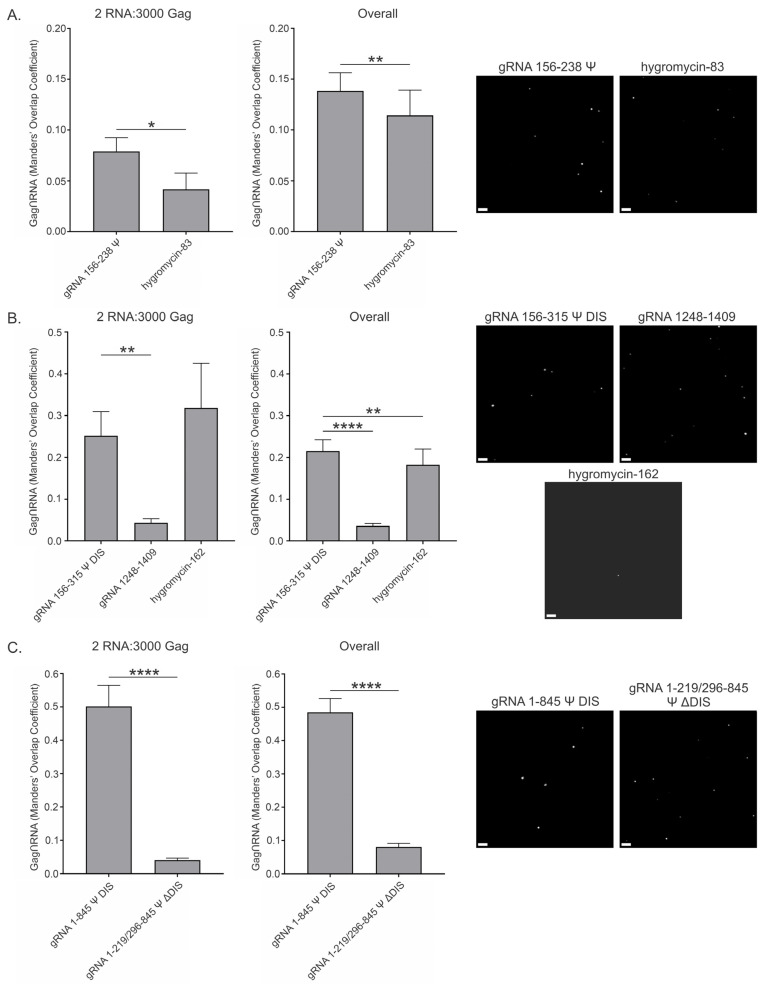
RSV Gag-RNA colocalization within 2.5 μM Gag condensates. Manders’ Overlap Coefficients for Gag∩RNA for each of the three RNA length groups ((**A**), short; (**B**), medium; (**C**), long) are displayed; both 2 RNA:3000 Gag (left panel) and the Overall (right panel) values are shown. Values are displayed as mean values ± S.E.M. (n ≥ 9 for 2 RNA:3000 Gag; n ≥ 39 for Overall). Statistical significance was determined by Mann–Whitney test (**A**,**C**) or Kruskal–Wallis test with Dunn’s post-hoc test (**B**) (****, *p* ≤ 0.0001; **, *p* ≤ 0.01; *, *p* ≤ 0.05). Representative images of RSV Gag and RNA colocalization (white) are shown. All images are of 2 RNA:3000 Gag; scale bars = 2 μm.

**Figure 7:**
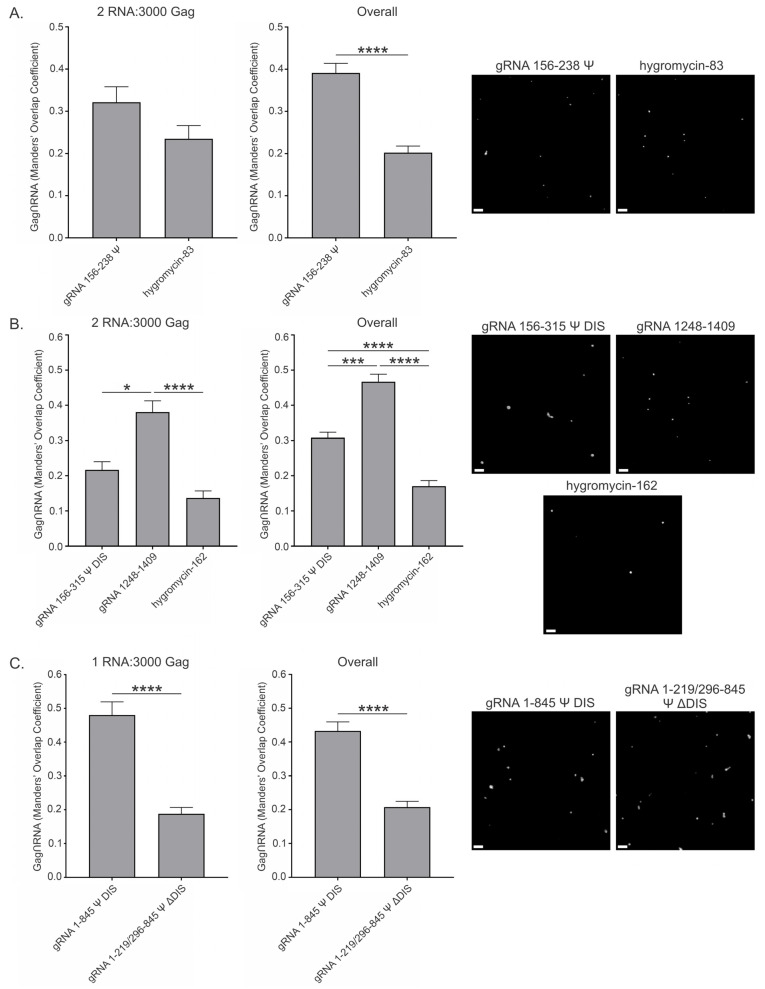
RSV Gag-RNA colocalization within 10 μM Gag condensates. Manders’ Overlap Coefficients for Gag∩RNA for each of the three RNA size groups ((**A**), short; (**B**), medium; (**C**), long) are displayed; both 2 (or 1) RNA:3000 Gag (left panel) and the Overall (right panel) values are shown. Values are displayed as mean values ± S.E.M. [n ≥ 10 for 2 (or 1) RNA:3000 Gag; n ≥ 20 for Overall]. Statistical significance was determined by Mann–Whitney test (**A**,**C**) or Kruskal–Wallis test with Dunn’s post-hoc test (**B**) (****, *p* ≤ 0.0001; ***, *p* ≤ 0.001; *, *p* ≤ 0.05). Representative images of RSV Gag and RNA colocalization (white) are shown. All images are of 2 (or 1) RNA:3000 Gag; scale bars = 10 μm.

Condensation is mediated by complex, multivalent interactions of varying types (specific/nonspecific), strengths (weak/intermediate/strong), and distances (short/long). Therefore, the effects on area and intensity observed in these in vitro assays could be due to direct interactions between Gag and RNAs, or the presence of RNAs could influence Gag condensation due to their effects on the assay conditions or environment. To better understand how each RNA could exert its influence, we examined the proportion of Gag that was colocalized with each RNA using signal-based colocalization. As with the area and intensity data above, colocalization was assessed at both 2.5 μM (Figure 6 and Figure S3) and 10 μM (Figure 7 and Figure S4) Gag. The yeast tRNA and 20U RNAs were omitted from all further assays, as the fluorescence labeling method was not suitable for these RNAs.

Both short RNAs examined—gRNA 156–238 Ψ and hygromycin-83—had less than 10% colocalization with Gag (7.9% ± 1.4% and 4.2% ± 1.6%, respectively) at 2.5 μM and 2 RNA:3000 Gag (Figure 6A and Table 5). These amounts were increased somewhat in the Overall data (13.8% ± 1.8% and 11.4% ± 2.5%, respectively) (Table 5 and Table S8), but were still among some of the lowest values observed. In both cases, significantly more Gag was colocalized with Ψ-containing gRNA 156–238 Ψ than with hygromycin-83, but it was still somewhat surprising to us that gRNA 156–238 Ψ exhibited such a low percent of colocalization because it contains Ψ. When considering the reason(s) for these relatively low amounts of colocalization observed with these RNAs, one possibility is that their relatively small size allowed many RNAs to occupy the same condensate.

In support of this possibility, the medium Ψ-containing RNA, gRNA 156–315 Ψ DIS, had roughly three times the amount of Gag colocalized with it at 2 RNA:3000 Gag (25.2% ± 5.8%) compared to gRNA 156–238 Ψ at 2.5 μM Gag (Figure 6B and Table 5). The larger size of this RNA may therefore limit the number of RNAs able to occupy each condensate, resulting in more condensates with RNA. This degree of colocalization was significantly higher than gRNA 1248–1409 (4.3% ± 1.0%) at the 2 RNA copy number; however, the proportion of Gag colocalized with hygromycin-162 was, perhaps surprisingly, higher (31.8% ± 10.7%), yet also more variable. As hygromycin-162 was shown to drastically decrease condensate area and intensity (likely due to charge-mediated resolubilization, as mentioned above) (Figure 2D), this high and variable degree of colocalization may be an artifact related to the low amount of condensed Gag present. All values for the medium RNAs were lower in the Overall data, reducing the value for hygromycin-162 (18.3% ± 3.8%) to below that of gRNA 156–315 Ψ DIS (21.6% ± 2.7%) and resulting in a significantly higher value for gRNA 156–315 Ψ DIS compared to hygromycin-162 and gRNA 1248–1409 (3.6% ± 0.6%) (Table 5 and Table S8).

When considering the long RNAs, the considerably higher percent colocalization values for gRNA 1–845 Ψ DIS at both 2 RNA:3000 Gag (50.1% ± 6.4%) and in the Overall data (48.5% ± 4.1%) further suggest that RNA size limited the quantity of RNAs permissible in each condensate (Figure 6C, Table 5 and Table S8). However, gRNA 1–219/296–845 Ψ ΔDIS did not follow this trend, instead displaying levels of colocalization at 2 RNA:3000 Gag (4.1% ± 0.6%) and in the Overall data (8.1% ± 1.1%) much more like those of gRNA 156–238 Ψ. This finding suggests that there must be another reason for the observed differences in Gag-RNA colocalization.

Notably, the colocalization values for gRNA 1–219/296–845 Ψ ΔDIS were also lower than those of gRNA 156–315 Ψ DIS. Both gRNA 156–315 Ψ DIS and gRNA 1–845 Ψ DIS contain the DIS (nt 258–274, Figure 1A), which forms a kissing-loop structure between RNAs resulting in a loose dimer [48,50]. In the case of gRNA 1–845 Ψ DIS, which also contains the DLS, this interaction is further stabilized. Taken together, these observations suggest that RNA dimerization may be important for Gag-RNA complex formation or condensate subunit architecture, as recently shown for an RNA dimer structure important for the formation of RNA granules in *Drosophila* [82].

In striking contrast to 2.5 μM Gag, considerably more colocalization was observed with the short RNAs at 10 μM Gag (Figure 7A, Table 5 and Table S8). Values for gRNA 156–238 Ψ and hygromycin-83 at 2 RNA:3000 Gag (32.1% ± 3.7% and 23.5% ± 3.1%, respectively) and in the Overall data (39.1% ± 2.3% and 20.2% ± 1.5%, respectively) continued to exhibit a preference for the Ψ-containing RNA. Interestingly, gRNA 1248–1409 displayed a marked increase in colocalization at 10 μM Gag for both 2 RNA:3000 Gag (38.0% ± 3.2%) and in the Overall data (46.7% ± 2.1%), resulting in a statistically significant difference compared to the other medium-length RNAs, gRNA 156–315 Ψ DIS (21.7% ± 2.3%, 30.8% ± 1.6%) and hygromycin-162 (13.7% ± 2.0%, 17.1% ± 1.6%) (Figure 7B, Table 5 and Table S8). Notably, these two medium RNAs retained relatively similar colocalization values to those at 2.5 μM Gag.

The colocalization value for gRNA 1–845 Ψ DIS at 1 RNA:3000 Gag (48.0% ± 3.9%) was not appreciably different than that at 2.5 μM Gag (Figure 7C and Table 5). The Overall value for this RNA (43.3% ± 2.7%) was also relatively similar (Table 5 and Table S8). In contrast, the values for gRNA 1–219/296–845 Ψ ΔDIS (18.8% ± 1.8%, 20.7% ± 1.7%) did increase by about two-fold. It is interesting that only the RNAs that displayed lower colocalization values at 2.5 μM Gag had an increased proportion of Gag colocalized with them at this higher protein concentration. One possibility for this finding is that the increased protein concentration promoted the coalescence of numerous smaller condensates, resulting in fewer condensates and therefore a higher proportion of condensates containing these RNAs.

Taken together, these results suggest that in vitro colocalization is not a prerequisite for population-level modulation of condensate abundance. Additionally, the data indicate a role for gRNA dimerization in Gag-gRNA colocalization, as evidenced by similar amounts of Gag colocalization with gRNA 156–315 Ψ DIS and gRNA 1–845 Ψ DIS at both protein concentrations.

### 3.7. Three-Dimensional Spatial Organization of Gag-RNA Co-Condensates

The data up to this point indicated how the various RNAs used in this study affected the area and intensity of Gag condensates as well as what proportion of Gag colocalized with each RNA, although the spatial organization of Gag and RNA within condensates was not yet examined. To determine condensate architecture, we acquired confocal z-stacks of condensates, then used Imaris Image Analysis Software to address this question.

**Figure 8:**
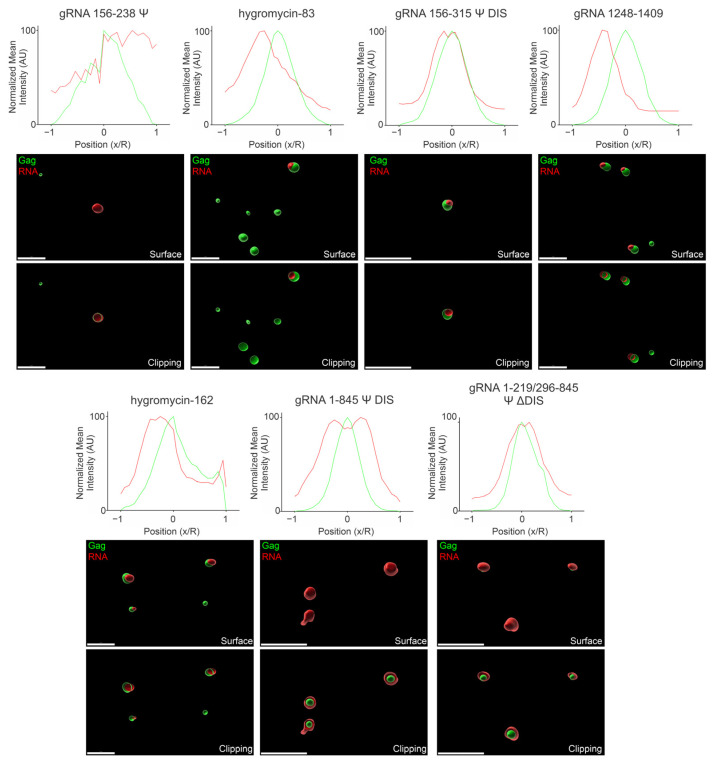
Three-dimensional organization of 2.5 μM Gag-RNA co-condensates, 2 RNA:3000 Gag. Fluorescent signal profiles of RSV Gag (green) and RNA (red) are shown, depicting various levels of signal peak overlap for 2 RNA:3000 Gag. Representative images of 3D organization of Gag (green)-RNA (red) condensates were generated using the surface rendering function in Imaris Image Analysis Software (Oxford Instruments, Abingdon, UK). Top images are of the surface and bottom images are orthogonal clipping planes through the condensates demonstrating internal organization. All images are of 2 RNA:3000 Gag; scale bars = 2 μm.

**Figure 9:**
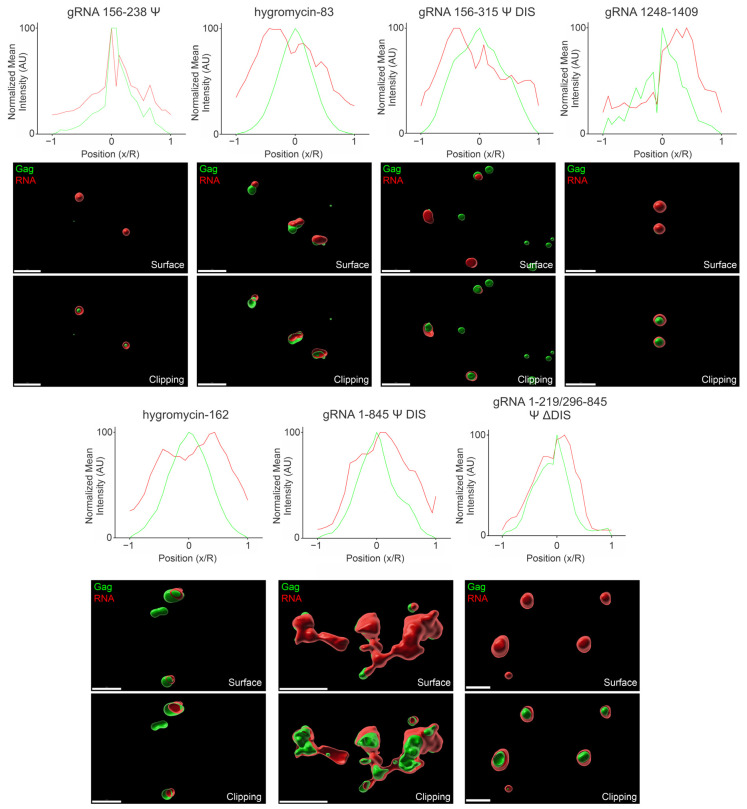
Three-dimensional organization of 10 μM Gag-RNA co-condensates, 2 (or 1) RNA:3000 Gag. Fluorescent signal profiles of RSV Gag (green) and RNA (red) are shown, depicting various levels of signal peak overlap for 2 RNA:3000 Gag (gRNA 1–845 Ψ DIS and gRNA 1–219/296–845 Ψ ΔDIS are 1 RNA:3000 Gag). Representative images of 3D organization of Gag (green)-RNA (red) condensates were generated using the surface rendering function in Imaris Image Analysis Software (Oxford Instruments, Abingdon, UK). The top images are of the surface and bottom images are orthogonal clipping planes through the condensates demonstrating internal organization. All images are of 2 RNA:3000 Gag, except for gRNA 1–845 Ψ DIS and gRNA 1–219/296–845 Ψ ΔDIS, which are 1 RNA:3000 Gag; scale bars = 2 μm.

Firstly, we generated surface renderings of the Gag and RNA signals to determine where each was located in relation to another (i.e., adjacent, overlapping, one enclosed within the other, etc.). Next, we examined the distribution of signal within these condensates by line profile to determine if the areas corresponding to the highest Gag or RNA signal overlapped. Due to the variability in condensate size and intensity, we applied a normalization to allow for their comparison, the details of which are described in Section 2. As with the previous figures, we assessed condensates at both 2.5 μM and 10 μM Gag, and at all four ratios of RNA:Gag (0.5/1/2/4:3000). Signal profiles for 2 RNA:3000 Gag (Figure 8 and Figure 9) were placed in the main text and the remainder are provided in Figures S5–S7.

When comparing the arrangement of Gag-gRNA 156–238 Ψ and Gag–hygromycin-83 co-condensates at 2.5 μM Gag and 2 RNA:3000 Gag (Figure 8), there was a noticeable difference. The Gag-gRNA 156–238 Ψ co-condensates displayed closely overlapping signal profiles in the region of the Gag signal peak, indicating the coexistence/comingling of Gag and RNA. This finding was supported by the surface and clipping images, which depicted Gag and RNA foci of relatively similar size overlapping with a small amount of additional RNA signal encircling the area of Gag-gRNA 156–238 Ψ coexistence. The Overall data agreed well with these findings, with the signal profiles once again closely overlapping in the region of the Gag signal peak (Figures S5 and S7). In contrast, the Gag–hygromycin-83 co-condensates displayed offset Gag and RNA signal peaks and only partial overlap in the surface and clipping images at 2 RNA:3000 Gag. This offset was somewhat reduced in the Overall profile, as all other RNA ratios tested contain higher levels of RNA signal corresponding to the Gag signal peak (Figures S5 and S7). The reason that the offset was greater at 2 RNA:3000 Gag is unclear, but these findings may also provide further insight into the low level of colocalization observed in Figure 6.

Similar to Gag-gRNA 156–238 Ψ co-condensates, Gag-gRNA 156–315 Ψ DIS co-condensate signal peaks closely overlapped at 2 RNA:3000 Gag and Overall, suggesting close association between Gag and this RNA. Surface and clipping images also depicted a high degree of overlap, if not as close as seen with gRNA 156–238 Ψ. These findings are consistent with the colocalization data shown in Figure 6 and are not surprising given the presence of Ψ in this construct. One potential explanation for the slight deviation observed may be that the RNA regions distal to Ψ may not be capable of packing as well into these relatively small (<1 μm) Gag condensates due either to size or charge density. Interestingly, the non-Ψ-containing gRNA 1248–1409 exhibited a similar, but slightly greater, signal peak offset and a less complete overlap in the surface and clipping images to that of hygromycin-83. However, in contrast to that RNA, gRNA 1248–1409 retained this offset with the other RNA ratios and in the Overall data (Figures S5 and S7). These observations, combined with Figure 2, Figure 3 and Figure 6 argue that this virally derived RNA exerts its effects primarily via longer-range electrostatic interactions that serve to space out Gag molecules within condensates. Taken in the context of the entire gRNA, such long-range interactions that reduce the close packing of Gag may be important for retaining mobility of Gag within condensates and ensuring irreversible aggregates do not form. These additional non-Ψ contacts may also ensure a critical mass of Gag is carried to the plasma membrane with Gag-gRNA complexes, facilitating rapid virion assembly. Also consistent with previous experiments, hygromycin-162 exhibited closely overlapping signal peaks and a fair amount of overlap in the surface and clipping images for both 2 RNA:3000 Gag and Overall (Figure 8, Figures S5 and S7).

Both gRNA 1–845 Ψ DIS and gRNA 1–219/296–845 Ψ ΔDIS displayed interesting morphologies via surface and clipping images, wherein Gag appeared to be encircled by RNA. In a similar manner to gRNA 156–315 Ψ DIS, the additional RNA length in these constructs may partially explain this phenomenon. A slight decrease in RNA in the center of these co-condensates is reflected in the signal profiles, consistent with the bulk of the RNA protruding from the central region containing Gag. This effect was more pronounced in gRNA 1–845 Ψ DIS, possibly due to increased RNA length driven by RNA-RNA contacts and dimerization mediated by the DIS and DLS.

Increasing the protein concentration to 10 μM Gag resulted in little change in the Gag-gRNA 156–238 Ψ signal peak overlap profile, suggesting similarly close association between these components (Figure 9, Figures S6 and S7). However, the surface and clipping images display additional RNA signal encircling the region of Gag-gRNA 156–238 Ψ coexistence compared to the lower protein concentration. This additional RNA surrounding Gag may help to offset the positive charge provided by Gag, allowing more Gag to pack into a similar area. Hygromycin-83 exhibited a noticeable offset in signal peaks and only partial overlap in the images, similar to that seen at the lower protein concentration. In contrast, however, this offset was retained in the Overall data as this overlap also existed at the other RNA ratios (Figures S6 and S7). Also in contrast to that seen at the lower protein concentration, there was a noticeable offset in the Gag-gRNA 156–315 Ψ DIS co-condensate signal peaks at 2 RNA:3000 Gag. However, the Overall data more closely overlaps (Figure S7).

Notably, while gRNA 1248–1409 still displayed offset signal peaks, the offset appeared lessened at this protein concentration. Additionally, the images reveal additional RNA signal encircling Gag; this change in condensate architecture may explain why there is a striking increase in colocalization with this construct at 10 μM Gag. In contrast to the lower protein concentration, hygromycin-162 appeared to be somewhat excluded from the Gag signal peak and displayed only partial overlap in the images. This change in architecture also aligns well with the lower colocalization observed in Figure 7.

Similar to what was observed with 2.5 μM Gag, both gRNA 1–845 Ψ DIS and gRNA 1–219/296–845 Ψ ΔDIS appeared to encircle Gag condensates and exhibited closely overlapping signal profiles at 1 RNA:3000 Gag and in the Overall data. Strikingly, gRNA 1–845 Ψ DIS appeared to bridge Gag-RNA co-condensates together, in contrast to any other RNA, forming clusters of Gag condensates surrounded by RNA. This phenomenon is likely due to the existence of stable RNA dimers mediated by the DIS and DLS sequences, which caused bridging of Gag condensates along the dimer. In contrast, gRNA 1–219/296–845 Ψ ΔDIS did not connect condensates together and instead may restrict condensate size when viewed in the context of Figure 4 and Figure 5.

### 3.8. Förster Resonance Energy Transfer (FRET) Analysis of Gag-RNA Co-Condensates

**Figure 10:**
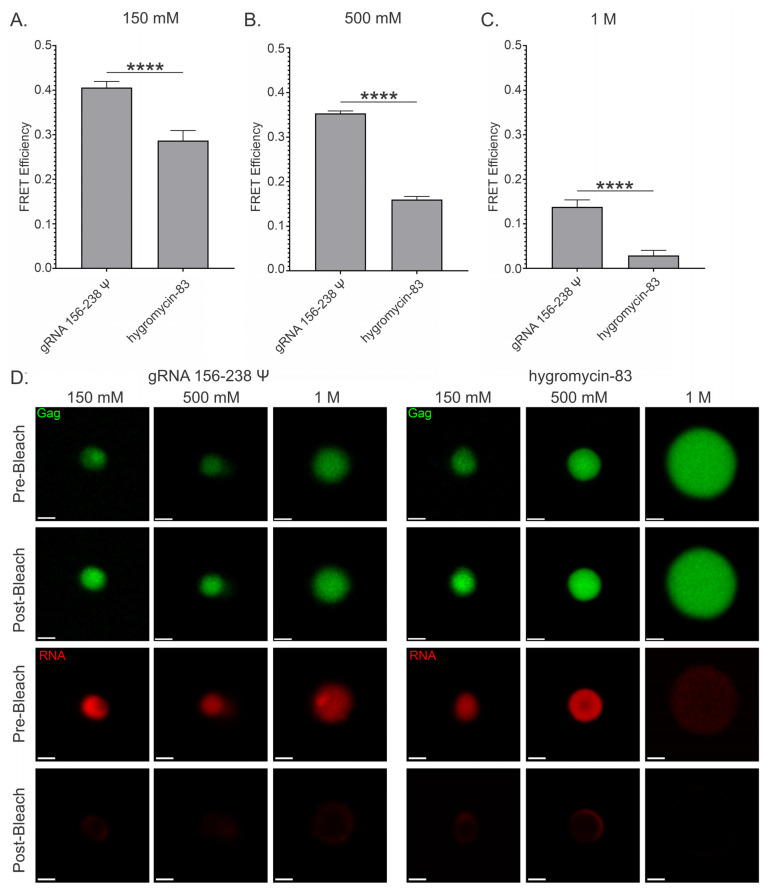
Förster Resonance Energy Transfer (FRET) of Gag-RNA co-condensates. (**A**–**C**) Decreasing FRET efficiency of Gag-RNA co-condensates in the presence of increasing concentrations of NaCl (A, 150 mM; B, 500 mM; C, 1 M) demonstrates the contribution of electrostatic interactions to close association of Gag and RNAs. Ψ-containing gRNA 156–238 Ψ, which interacts specifically with Gag, is more resistant to electrostatic disruption. Values are displayed as mean values ± S.E.M. (n ≥ 20). Statistical significance was determined by Mann–Whitney test (****, *p* ≤ 0.0001). (**D**) Representative pre- and post-acceptor bleach images for all conditions tested for both Gag (green) and RNA (red) are shown and highlight differences in condensate morphologies with these two RNAs; scale bars = 2 μm.

As mentioned, BMCs form via complex and varied multivalent interactions between proteins and nucleic acids. Our work thus far has, in agreement with many prior studies on varied protein–RNA systems, suggested that both sequence-driven, specific interactions and charge-based, electrostatic interactions contribute to the phenomenon of RSV Gag-RNA condensates. In the context of condensates, intermolecular interactions may be further stabilized and components brought closer together by the dense environment within them. As we know that the Gag-Ψ interaction is critical for retroviral genome selection and packaging, we wanted to assess whether there was any difference between the distance of the interaction between Gag and Ψ-containing RNAs compared to nonviral RNAs in condensates. While the colocalization data are compelling, the resolution limit of the microscope prevents one from assessing direct interaction between Gag and RNA. Thus, we utilized the advanced imaging technique Förster Resonance Energy Transfer (FRET), to compare FRET efficiencies between Gag and either the viral, Ψ-containing gRNA 156–238 Ψ or a nonviral RNA of similar length, hygromycin-83 (Figure 10). It was previously demonstrated that HIV-1 Gag binds both Ψ and non-Ψ RNAs with similar affinities at physiologic salt concentrations (150 mM), and that both HIV-1 and RSV Gag binding to Ψ-containing RNAs is more resistant to ionic disruption [40,41]. Therefore, we performed these experiments at three different concentrations of NaCl (150 mM, 500 mM, and 1 M) to determine whether increased ionic strength would decrease FRET efficiency between Gag and these RNAs in the context of condensates.

The Ψ-containing gRNA 156–238 Ψ exhibited significantly greater FRET efficiency than hygromycin-83 at all concentrations of NaCl tested (150 mM: 0.406 ± 0.014 vs 0.287 ± 0.006, *p* ≤ 0.0001; 500 mM: 0.353 ± 0.016 vs 0.160 ± 0.023, *p* ≤ 0.0001; 1M: 0.138 ± 0.007 vs 0.029 ± 0.011, *p* ≤ 0.0001), indicating that Gag and gRNA 156–238 Ψ interact more closely than Gag and hygromycin-83 in condensates (Figure 10A–C). Furthermore, the ratio of the FRET efficiencies of gRNA 156–238 Ψ and hygromycin-83 increased with higher salt (1.42:1, 2.21:1, and 4.76:1, respectively), indicating that the Gag-Ψ interaction was recalcitrant to increased ionic strength in agreement with the prior studies mentioned above [40,41]. Interestingly, increasing salt also had a much less drastic effect on condensate morphology in the presence of gRNA 156–238 Ψ, suggesting that this RNA, and by extension, the Gag-Ψ interaction, may effectively help to stabilize condensate morphology against changes in ionic strength (Figure 10D).

Together, these findings suggest that Gag is better able to discriminate between Ψ and non-Ψ RNAs under conditions that mimic the crowded intracellular environment, compared to in solution, reinforcing the importance of the Gag-Ψ interaction in the formation of stable BMCs.

### 3.9. Fluorescence Recovery After Photobleaching (FRAP) of Gag-RNA Co-Condensates

As mentioned previously, a key function of RNA within condensates is to modulate protein–protein interactions and prevent pathological aggregation [72,78,79,80,81]. While Gag is believed to form weak dimers in solution that are stabilized by nucleic acid binding and further multimerize during membrane binding preceding virion assembly, it is possible that the densely packed environment within the in vitro condensates strongly stabilizes these Gag-Gag interactions, limiting mobility. We have previously published fluorescence recovery after photobleaching (FRAP) results analyzing RSV Gag condensates in cells [2], which demonstrated that Gag within these condensates remains mobile with a very fast exchange rate.

**Figure 11:**
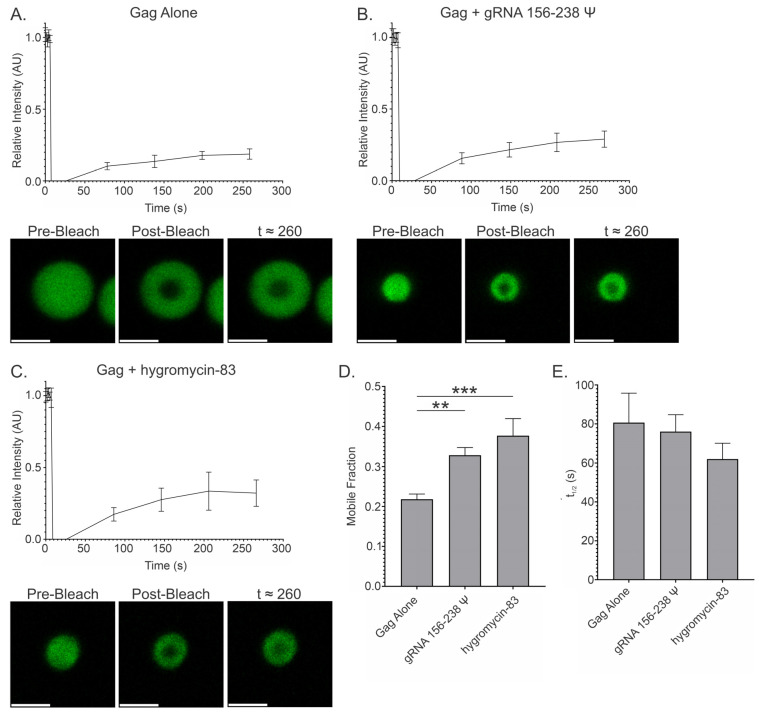
Fluorescence recovery after photobleaching (FRAP) of Gag-RNA co-condensates. (**A**–**C**) FRAP analysis of Gag alone, Gag + gRNA 156–238 Ψ, and Gag + hygromycin-83 co-condensates (n ≥ 10). Recovery plots (top) and representative images of pre-bleach, post-bleach, and post-recovery conditions (bottom) are displayed; scale bars = 2 μm. Panels (**D**) and (**E**) display condensate mobile fraction and t_1/2_ (s) values, respectively. The presence of either gRNA 156–238 Ψ or hygromycin-83 resulted in significantly increased mobile fractions and somewhat reduced t_1/2_ (s) values compared to Gag alone. Values are displayed as mean values ± S.E.M. (n ≥ 10). Statistical significance was determined by Kruskal–Wallis test with Dunn’s post-hoc test (***, *p* ≤ 0.001; **, *p* ≤ 0.01).

As a complement to both our FRET and previously published in-cell data [2], we next compared the effect of a Ψ-containing viral RNA (gRNA 156–238 Ψ) and a nonviral RNA (hygromycin-83) on the mobility of Gag within our in vitro condensates (Figure 11). In the absence of RNA, we indeed observed limited mobility of Gag, as indicated by a relatively low mobile fraction (21.8%) and comparably long t_1/2_ (80.67 s) (Figure 11A,D,E). Interestingly, this mobile fraction was strikingly similar to what we observed previously in the nucleus of cells (25.0% ± 0.02), suggesting that our methodology effectively mimics this environment. However, the t_1/2_ was considerably shorter (1.25 ± 0.22 s) in the nucleus, likely due to the presence of nucleic acids and other interacting proteins that could modulate strong Gag-Gag interactions [2,24]. As expected, the presence of gRNA 156–238 Ψ or hygromycin-83 within condensates significantly increased protein mobility compared to condensates containing only Gag, as evidenced by increased mobile fraction values (32.8% and 37.7%, respectively; Figure 11B–E). Although the mean half time of recovery (t_1/2_) was decreased in the presence of gRNA 156–238 Ψ or hygromycin-83 (76.0 and 61.97 s, respectively), the recovery times were longer than in cells due to the differences in the composition of the surrounding environment. It is notable, however, that hygromycin-83 resulted in a marginally greater mobile fraction and shorter t_1/2_ compared to gRNA 156–238 Ψ, suggesting that electrostatic interactions with non-Ψ RNAs are arguably more important for retaining mobility.

## 4. Discussion

After many productive years of research aimed at developing therapeutic approaches to combat retroviral infection, there remain areas that are as yet untapped for clinical intervention. The pioneering observations that the RSV and HIV-1 Gag proteins colocalize with their cognate USvRNAs at transcription sites [70,83], participate in BMC formation [2,3], and potentially interact with numerous nuclear proteins [24,84,85,86,87,88,89] underscore the value of studying retroviral Gag-containing condensates. The large set of nuclear protein interactors identified suggests that the composition of RSV Gag-containing condensates within infected cells is complex, and likely dynamic, over the course of the condensates’ lifetimes. Additionally, the presence of numerous cellular and viral RNA species of varied lengths and conformations at transcription sites and the important role of RNA in the regulation of BMCs adds to this complexity.

As a first step towards unraveling the mechanisms underlying Gag-RNA BMC formation, this manuscript characterized the effect(s) of viral and nonviral RNAs on the biophysical properties of RSV Gag BMCs using in vitro methods. While simplified compositionally, the in vitro methodology utilized makes use of physiologic salt concentration and molecular crowding to effectively mimic the dense nuclear environment. As mentioned throughout this work, there are often disparities between modes of interaction in dilute solution versus conditions using crowding agents. As such, this work represents an important step forward in the study of Gag-RNA interaction and the study of this component of retroviral assembly by investigating in a systematic manner how biomolecular condensation impacts, and is impacted by, the Gag-Ψ interaction and the formation of gRNA dimers mediated by the DIS.

In cellular BMC-forming proteins such as FUS, TDP-43, hnRNPA1, and G3BP1, RNA significantly alters both the propensity of these proteins to form condensates and condensate properties such as size, shape, viscosity/liquidity, surface tension, and composition [57,59,72,75,90,91,92,93,94]. Much of this work has been done using well-defined, artificial nucleic acids and simple anionic polymers (i.e., polyA/U/C/G), which fail to capture the contributions of sequence and structure to protein–RNA and RNA-RNA interactions [59,75,95,96]. Nonetheless, such artificial RNAs have been utilized to effectively demonstrate the importance of RNA and the varied roles it plays in regulating condensates. RNA can promote condensation by serving as a scaffold, which balances intra- and inter-protein interactions that limit molecular mobility [28,72,95,96,97]. Self-assembling proteins such as TDP-43 and hnRNPA1 have been shown to utilize RNA binding to prevent pathological self-multimerization [72,78,79,80,81]. RNA binding can also promote condensation by inducing conformational changes in protein components, as is the case for G3BP1 [59]. In other cases, RNA can antagonize condensate formation, as is the case for transcriptional super-enhancer complexes, which are dissolved in the presence of abundant newly-synthesized RNA [4,98]. Interestingly, the dense environment within condensates can also affect the orientation of proteins and RNA, resulting in altered conformations, multimerization states, and points of contact between molecules [81].

**Figure 12:**
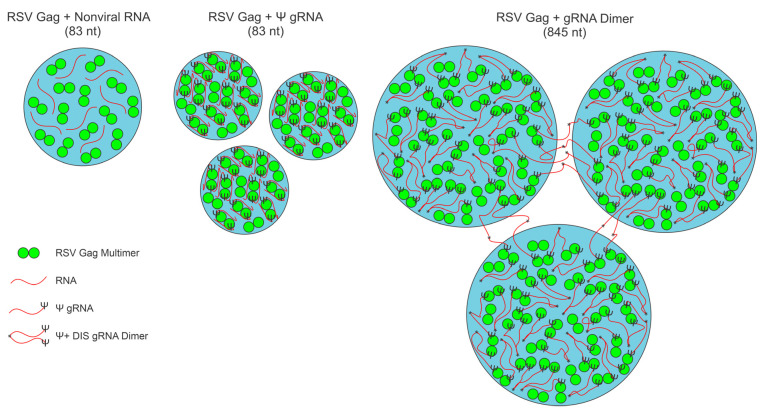
RSV Gag-RNA condensate models. Illustration showing examples of RSV Gag-RNA co-condensates depicted to highlight the differential roles of nonviral and viral RNA containing Ψ (gRNA 156–238 Ψ), and Ψ + DIS (gRNA 1–845 Ψ DIS) on Gag BMC architecture. Nonviral RNAs primarily functioned to space out Gag molecules, resulting in loosely packed condensates. The short viral RNA containing Ψ formed relatively small, densely packed Gag condensates. Viral gRNAs containing Ψ and DIS promoted the formation of large, densely packed Gag condensates. Stabilization of the gRNA dimer by the presence of Ψ, DIS, and DLS in gRNA 1–845 Ψ DIS formed interconnected clusters of condensates at 10 μM, possibly connected by dimers of RNAs between adjacent condensates.

We performed in vitro condensation assays using fluorescently labeled Gag and RNAs (where possible) to examine how each RNA affected the abundance of condensed RSV Gag. At the lower protein regime (2.5 μM Gag), there was generally higher sensitivity to the presence of RNAs, regardless of source (viral or nonviral). Importantly, these data (Figure 2, Figure 3, Figure 4 and Figure 5) highlight the importance of both specific (i.e., Ψ) and nonspecific (i.e., electrostatic) interactions in influencing condensation (Figure 12), and the role of local protein concentration on condensates’ susceptibility to these influences. In general, Ψ-containing RNAs had a stabilizing effect on BMCs, likely mediated by stable complex formation, and this effect was enhanced in the presence of the DIS. Importantly, Gag-Gag multimers and DIS-mediated RNA dimers can work synergistically to this end. In contrast, electrostatically driven Gag-RNA interactions appear to function more similarly to cellular RNA binding proteins, such as transcription super-enhancer complexes [4], promoting or antagonizing condensation depending on local protein concentration and condensate architecture.

The inclusion of two separate Gag protein concentrations (2.5 μM and 10 μM) was critical for elucidating effects of RNA size and structure (Figure S8) on RSV Gag condensates. Likewise, the inclusion of Ψ-containing RNAs of multiple lengths, as well as those containing or lacking the DIS, provide novel insights into the role(s) of these viral RNA features in condensate formation and suggest that gRNA dimerization contributes to multiple steps in retroviral genome packaging. A model explaining how each of the RNAs tested affected Gag condensate formation is shown in Figure 12.

Selective 2′-hydroxyl acylation analyzed by primer extension (SHAPE) was used in a prior in vitro study of the Gag-gRNA interaction to identify six RNA residues within the 5′-untranslated region (5′ UTR) of RSV gRNA that are important for the Gag-gRNA interaction [48]. The median of the SHAPE reactivity ranges provided in that manuscript were used as restraints to generate in silico predictions of the secondary structures of the Ψ-containing RNAs presented in the current manuscript (Figure S8). Notably, gRNA 1–219/296–845 Ψ ΔDIS lacks two of the six residues, which could contribute to some degree towards the differences between this RNA and other Ψ-containing RNAs. Additionally, the predicted secondary structure of gRNA 1–219/296–845 Ψ ΔDIS does appear to be altered by the internal deletion when compared to gRNA 1–845 Ψ DIS. Therefore, this deletion could perturb RNA structure important for Gag-gRNA interactions.

Another interesting observation concerning these viral RNAs was that colocalization values for gRNA 156–315 Ψ DIS and gRNA 1–845 Ψ DIS did not vary much between protein concentrations, although those for gRNA 156–238 Ψ and gRNA 1–219/296–845 Ψ ΔDIS were more disparate (Figure 6 and Figure 7). Both gRNA 156–315 Ψ DIS and gRNA 1–845 Ψ DIS possess the DIS, arguing that dimerization (potentially only transient in the case of gRNA 156–315 Ψ DIS due to the lack of the DLS) may impart an advantage in terms of colocalization with Gag, likely due to intra-condensate subunit linkages. This possibility is supported by recent studies of RNP granules from *Drosophila melanogaster* and *Ashbya gossypii*, in which RNA sequence and structure have been shown to be important for condensate compositional specificity [82,99,100]. Notably, a similar kissing-loop dimerization interaction exists between *Drosophila oskar* RNAs, and dimerization promotes BMC formation with its cognate RNA binding protein Staufen [101]. In our study, gRNA 1–845 Ψ DIS, which contains all of the elements needed to form a stable dimer (Ψ, DIS, and DLS), formed clustered condensates at 10 μM Gag that appeared to be linked primarily by inter-condensate RNA interactions, suggesting a similar role in scaffolding condensates as *oskar* RNA dimers (Figure 9 and Figure 12). Taken together, our data suggest that the mesoscale bridging of Gag condensates by RNA dimers could also be occurring on the microscale, below the limit of detection by the approaches utilized. This possibility could be examined by additional experimental approaches such as super-resolution and atomic force microscopy.

The FRET and FRAP experiments also highlight the different but synergistic roles of Ψ-containing viral RNAs and nonviral RNAs in RSV Gag condensates, with the former appearing to stabilize architecture and the latter promoting condensate mobility (Figure 10 and Figure 11). When placed in the context of the cell, it seems likely that the interactions between Gag-gRNA complexes, nonviral RNAs, and potentially host proteins known to interact with Gag [24] help to keep these Gag-gRNA complexes mobile and of a reasonable size to pass through the nuclear pore *en route* to the plasma membrane for virion assembly. Further studies are warranted to fully characterize the complex nature of these RSV Gag-RNA condensates, but the methodology utilized and findings presented herein represent crucial steps in understanding and characterizing the effect(s) of RNAs of varied sources, sequences, and structures on RSV Gag BMCs. This work provides a framework for further study aimed at understanding the complex interactions between viral RNAs, nonviral RNAs, and RSV Gag in condensates, and the mechanism by which gRNA dimers promote Gag BMC formation, encapsidation, and virion assembly.

## Figures and Tables

**Table 1:** Log_2_ fold change in RSV Gag condensate area and intensity, 2.5 μM Gag and 2 RNA:3000 Gag.

RNA	Area (Log_2_ Fold Change)	Intensity (Log_2_ Fold Change)
gRNA 156–238 Ψ	0.691 ± 0.157	1.438 ± 0.148
hygromycin-83	2.515 ± 0.224	2.432 ± 0.239
Yeast tRNA	1.150 ± 0.095	1.092 ± 0.145
20U	−1.937 ± 0.821	−3.146 ± 0.865
gRNA 156–315 Ψ DIS	1.311 ± 0.200	2.764 ± 0.229
gRNA 1248–1409	1.420 ± 0.244	0.927 ± 0.292
hygromycin-162	−1.712 ± 0.678	−2.724 ± 0.805
gRNA 1–845 Ψ DIS	1.980 ± 0.162	3.486 ± 0.148
gRNA 1–219/296–845 Ψ ΔDIS	1.117 ± 0.137	0.927 ± 0.138

**Table 2:** Log_2_ fold change in RSV Gag condensate area and intensity, 2.5 μM Gag Overall.

RNA	Area (Log_2_ Fold Change)	Intensity (Log_2_ Fold Change)
gRNA 156–238 Ψ	0.762 ± 0.075	1.785 ± 0.090
hygromycin-83	1.833 ± 0.147	1.488 ± 0.150
Yeast tRNA	−0.167 ± 0.228	−0.653 ± 0.273
20U	−1.857 ± 0.377	−2.921 ± 0.403
gRNA 156–315 Ψ DIS	1.489 ± 0.107	2.813 ± 0.115
gRNA 1248–1409	1.416 ± 0.187	0.979 ± 0.230
hygromycin-162	−1.661 ± 0.281	−2.369 ± 0.290
gRNA 1–845 Ψ DIS	1.896 ± 0.095	3.439 ± 0.117
gRNA 1–219/296–845 Ψ ΔDIS	1.625 ± 0.090	1.552 ± 0.117

**Table 3:** Log_2_ fold change in RSV Gag condensate area and intensity, 10 μM Gag and 2 RNA:3000 Gag.

RNA	Area (Log_2_ Fold Change)	Intensity (Log_2_ Fold Change)
gRNA 156–238 Ψ	−0.245 ± 0.090	0.383 ± 0.112
hygromycin-83	0.256 ± 0.084	0.383 ± 0.191
Yeast tRNA	0.931 ± 0.035	0.528 ± 0.043
20U	1.036 ± 0.045	0.943 ± 0.118
gRNA 156–315 Ψ DIS	0.382 ± 0.061	0.491 ± 0.159
gRNA 1248–1409	−0.285 ± 0.189	−0.468 ± 0.090
hygromycin-162	0.022 ± 0.101	0.229 ± 0.206
gRNA 1–845 Ψ DIS *	0.439 ± 0.072	0.462 ± 0.154
gRNA 1–219/296–845 Ψ ΔDIS *	−0.374 ± 0.165	−0.220 ± 0.293

*, 1 RNA:3000 Gag.

**Table 4:** Log_2_ fold change in RSV Gag condensate area and intensity, 10 μM Gag Overall.

RNA	Area (Log_2_ Fold Change)	Intensity (Log_2_ Fold Change)
gRNA 156–238 Ψ	0.004 ± 0.053	0.816 ± 0.069
hygromycin-83	0.334 ± 0.048	0.455 ± 0.079
Yeast tRNA	0.732 ± 0.060	0.254 ± 0.060
20U	0.939 ± 0.031	1.029 ± 0.052
gRNA 156–315 Ψ DIS	0.281 ± 0.045	0.656 ± 0.096
gRNA 1248–1409	−0.085 ± 0.072	0.341 ± 0.069
hygromycin-162	−0.038 ± 0.046	0.233 ± 0.119
gRNA 1–845 Ψ DIS	0.610 ± 0.057	0.638 ± 0.094
gRNA 1–219/296–845 Ψ ΔDIS	−0.442 ± 0.108	−0.224 ± 0.187

**Table 5:** RSV Gag colocalization at 2.5 μM and 10 μM with viral and nonviral RNAs.

RNA	2.5 μM Gag		10 μM Gag	
	2 RNA:3000 Gag	Overall	2 RNA:3000 Gag	Overall
gRNA 156–238 Ψ	0.079 ± 0.014	0.138 ± 0.018	0.321 ± 0.037	0.391 ± 0.023
hygromycin-83	0.042 ± 0.016	0.114 ± 0.025	0.235 ± 0.031	0.202 ± 0.015
Yeast tRNA	N.D.	N.D.	N.D.	N.D.
20U	N.D.	N.D.	N.D.	N.D.
gRNA 156–315 Ψ DIS	0.252 ± 0.058	0.216 ± 0.027	0.217 ± 0.023	0.308 ± 0.016
gRNA 1248–1409	0.043 ± 0.010	0.036 ± 0.006	0.380 ± 0.032	0.467 ± 0.021
hygromycin-162	0.318 ± 0.107	0.183 ± 0.038	0.137 ± 0.020	0.171 ± 0.016
gRNA 1–845 Ψ DIS	0.501 ± 0.064	0.485 ± 0.041	0.480 ± 0.039	0.433 ± 0.027
gRNA 1–219/296–845 Ψ ΔDIS	0.041 ± 0.006	0.081 ± 0.011	0.188 ± 0.018	0.207 ± 0.017

N.D., not determined.

## Data Availability

Data are present within the primary article and in the Supplementary Materials.

## Supplementary Materials

The following supporting information can be downloaded at: https://www.mdpi.com/article/10.3390/v17010097/s1, Figure S1. Effect of RNA on 2.5 μM RSV Gag condensates, 0.5/1/4 RNA:3000 Gag; Figure S2. Effect of RNA on 10 μM RSV Gag condensates, 0.5/1/4 RNA:3000 Gag; Figure S3. RSV Gag-RNA colocalization within 2.5 μM RSV Gag condensates, 0.5/1/4 RNA:3000 Gag; Figure S4. RSV Gag-RNA colocalization within 10 μM RSV Gag condensates, 0.5/1/4 RNA:3000 Gag; Figure S5. Three-dimensional organization of 2.5 μM RSV Gag-RNA co-condensates, 0.5/1/4 RNA:3000 Gag; Figure S6. Three-dimensional organization of 10 μM RSV Gag-RNA co-condensates, 0.5/1/4 RNA:3000 Gag; Figure S7. Overall three-dimensional organization of RSV Gag-RNA co-condensates; Figure S8. Predicted secondary structure of RNAs; Table S1. 2.5 μM Gag; 0.5 RNA:3000 Gag; Table S2. 2.5 μM Gag; 1 RNA:3000 Gag; Table S3. 2.5 μM Gag; 4 RNA:3000 Gag; Table S4. 10 μM Gag; 0.5 RNA:3000 Gag; Table S5. 10 μM Gag; 1 RNA:3000 Gag; Table S6. 10 μM Gag; 4 RNA:3000 Gag; Table S7. Composite Factors; Table S8. Gag∩RNA Colocalization.

## Appendix A

156–238 Ψ: 5′ GAUCCUGCCCUCAUCCGUCUCGCUUAUUCGGGGAGCGGACGAUGACCCUAGUAGAGGGGGCUGCGGCUUAGGAGGGCAGAAGA 3′

156–315 Ψ DIS: 5′ AUCCUGCCCUCAUCCGUCUCGCUUAUUCGGGGAGCGGACGAUGACCCUAGUAGAGGGGGCUGCGGCUUAGGAGGGCAGAAGCUGAGUGGCGUCGGAGGGAGCUCUACUGCAGGGAGCCCAGAUACCCUACCGAGAACUCAGAGAGUCGUUGGAAGACGG 3′

1248–1409: 5′ GGACGUCACGAAUCUAAUGAGAGUUAUUUUAGGGCCUGCCCCAUAUGCCUUAUGGAUGGACGCUUGGGGAGUCCAACUCCAGACAGUUAUAGCGGCAGCCACUCGCGACCCCCGACACCCAGCGAACGGUCAAGGGCGGGGGGAACGGACUAAUUUGAAUCA 3′

1–845 Ψ DIS: 5′ GCCAUUUGACCAUUCACCACAUUGGUGUGCACCUGGGUUGAUGGCCGGACCGUUGAUUCCCUGACGACUACGAGCACCUGCAUGAAGCAGAAGGCUUCAUUUGGUGACCCCGACGUGAUAGUUAGGGAAUAGUGGUCGGCCACAGACGGCGUGGCGAUCCUGUCUCCAUCCGUCUCGUCUAUCGGGAGGCGAGUUCGAUGACCCUGGUGGAGGGGGCUGCGGCUUAGGGAGGCAGAAGCUGAGUACCGUCGGAGGGAGCUCUACUGCAGGGAGCCCAGAUACCCUACCGAGAACUCAGAGAGUCGUUGGAAGACGGGAAGGAAGCCCGACGACUGAGCAGUCCACCCCAGGCGUGAUUCUGGUCGCCCGGUGGAUCAAGCAUGGAAGCCGUCAUAAAGGUGAUUUCGUCCGCGUGUAAAACCUAUUGCGGGAAAACCUCUCCUUCUAAGAAGGAAAUAGGGGCCAUGUUGUCCCUCUUACAAAAGGAAGGGUUGCUUAUGUCUCCCUCAGACUUAUAUUCCCCGGGGUCCUGGGAUCCCAUUACCGCGGCGCUAUCCCAGCGGGCUAUGAUACUUGGGAAAUCGGGAGAGUUAAAAACCUGGGGAUUGGUUUUGGGGGCAUUGAAGGCGGCUCGAGAGGAACAGGUUACAUCUGAGCAAGCAAAGUUUUGGUUGGGAUUAGGGGGAGGGAGGGUCUCUCCCCCAGGUCCGGAGUGCAUCGAGAAACCAGCAACGGAGCGGCGAAUCGACAAAGGGGAGGAAGUGGGAGAAACAACUGUGCAGCGAGAUGCGAAGAUGGCGCCGGAGGAAACGGCCACACCUAAAACCGUUGGCACAUCCUGCUAU 3′

1–219/296–845 Ψ ΔDIS: 5′

GCCAUUUGACCAUUCACCACAUUGGUGUGCACCUGGGUUGAUGGCCGGACCGUUGAUUCCCUGACGACUACGAGCACCUGCAUGAAGCAGAAGGCUUCAUUUGGUGACCCCGACGUGAUAGUUAGGGAAUAGUGGUCGGCCACAGACGGCGUGGCGAUCCUGUCUCCAUCCGUCUCGUCUAUCGGGAGGCGAGUUCGAUGACCCUGGUGGAGGGGGCUAGAGAGUCGUUGGAAGACGGGAAGGAAGCCCGACGACUGAGCAGUCCACCCCAGGCGUGAUUCUGGUCGCCCGGUGGAUCAAGCAUGGAAGCCGUCAUAAAGGUGAUUUCGUCCGCGUGUAAAACCUAUUGCGGGAAAACCUCUCCUUCUAAGAAGGAAAUAGGGGCCAUGUUGUCCCUCUUACAAAAGGAAGGGUUGCUUAUGUCUCCCUCAGACUUAUAUUCCCCGGGGUCCUGGGAUCCCAUUACCGCGGCGCUAUCCCAGCGGGCUAUGAUACUUGGGAAAUCGGGAGAGUUAAAAACCUGGGGAUUGGUUUUGGGGGCAUUGAAGGCGGCUCGAGAGGAACAGGUUACAUCUGAGCAAGCAAAGUUUUGGUUGGGAUUAGGGGGAGGGAGGGUCUCUCCCCCAGGUCCGGAGUGCAUCGAGAAACCAGCAACGGAGCGGCGAAUCGACAAAGGGGAGGAAGUGGGAGAAACAACUGUGCAGCGAGAUGCGAAGAUGGCGCCGGAGGAAACGGCCACACCUAAAACCGUUGGCACAUCCUGCUAU 3′

hygromycin-83: 5′ AUGAAAAAGCCUGAACUCACCGCGACGUCUGUCGAGAAGUUUCUGAUCGAAAAGUUCGACAGCGUCUCCGACCUGAUGCAGCU 3′

hygromycin-162: 5′ AUGAAAAAGCCUGAACUCACCGCGACGUCUGUCGAGAAGUUUCUGAUCGAAAAGUUCGACAGCGUCUCCGACCUGAUGCAGCUCUCGGAGGGCGAAGAAUCUCGUGCUUUCAGCUUCGAUGUAGGAGGGCGUGGAUAUGUCCUGCGGGUAAAUAGCUGCGCC 3′

20U: UUUUUUUUUUUUUUUUUUUU

Yeast tRNA: Mixed population of tRNAs; see Invitrogen, #AM7119 for additional product details.
