# Supplementary material for: Role of the Psi Packaging Signal and Dimerization Initiation Sequence in the Organization of Rous Sarcoma Virus Gag-gRNA Co-Condensates
Source: Viruses. 2025 Jan 13;17(1):97. doi: 10.3390/v17010097 (PMC11769450; doi:10.3390/v17010097)
Supplement: Supplementary file 1 [file viruses-17-00097-s001.zip › viruses-3381771-supplementary.pdf]

viruses

MDPI

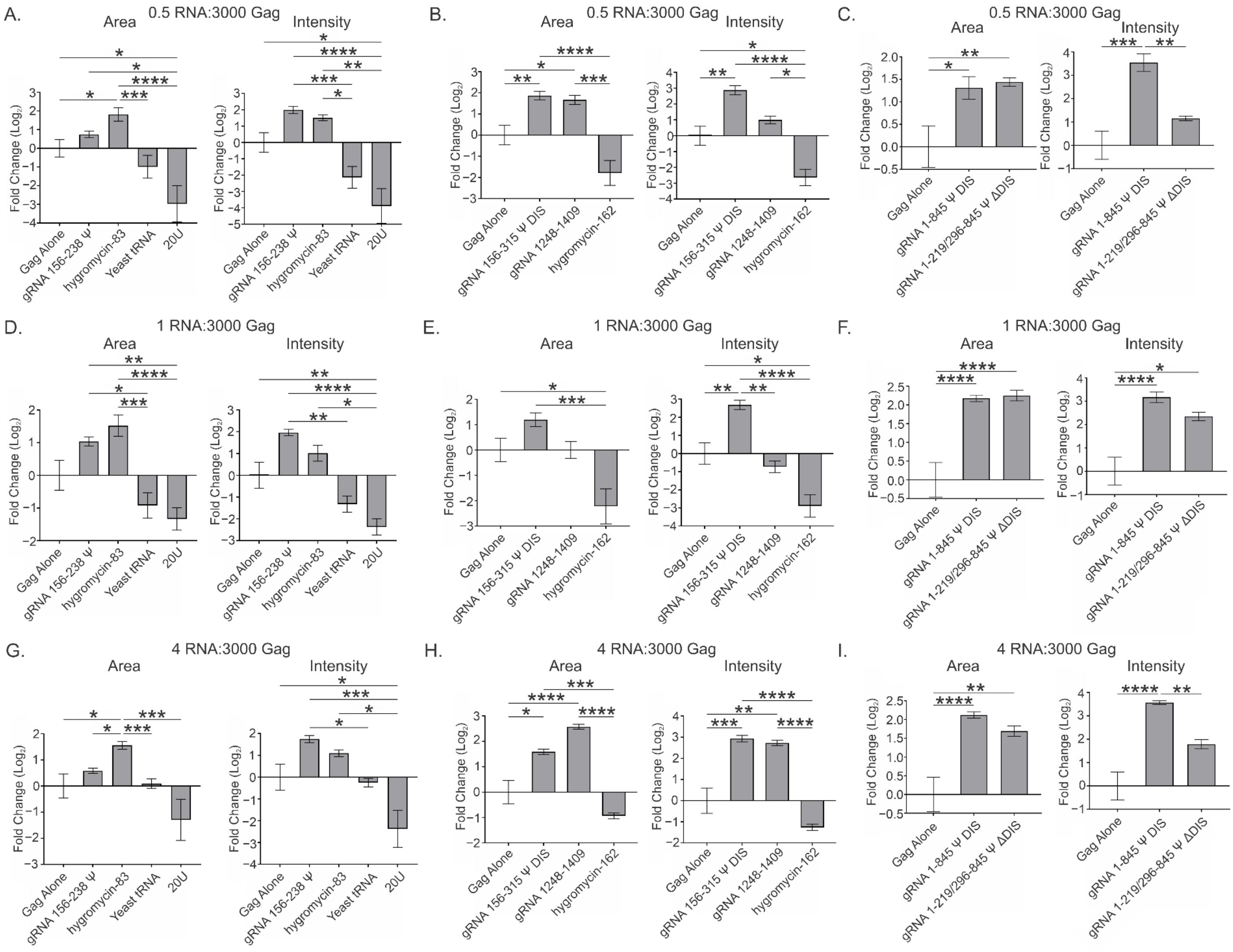

Figure S1. Effect of RNA on 2.5 µM RSV Gag condensates, 0.5/1/4 RNA:3000 Gag. Fold changes (Log2) in condensate area and intensity (left and right panels, respectively) for each of three ratios of RNA:3000 Gag (A-C, 0.5 RNA:3000 Gag; D-F, 1 RNA:3000 Gag; G-I, 4 RNA:3000 Gag) and three RNA length groups (A, D, G, short; B, E, H, medium; C, F, I, long) are displayed as mean values ± S.E.M. ($n \geq 9$). Statistical significance was determined by Kruskal–Wallis test with Dunn's post-hoc test (****, $p \leq 0.0001$; ***, $p \leq 0.001$; **, $p \leq 0.01$; *, $p \leq 0.05$).

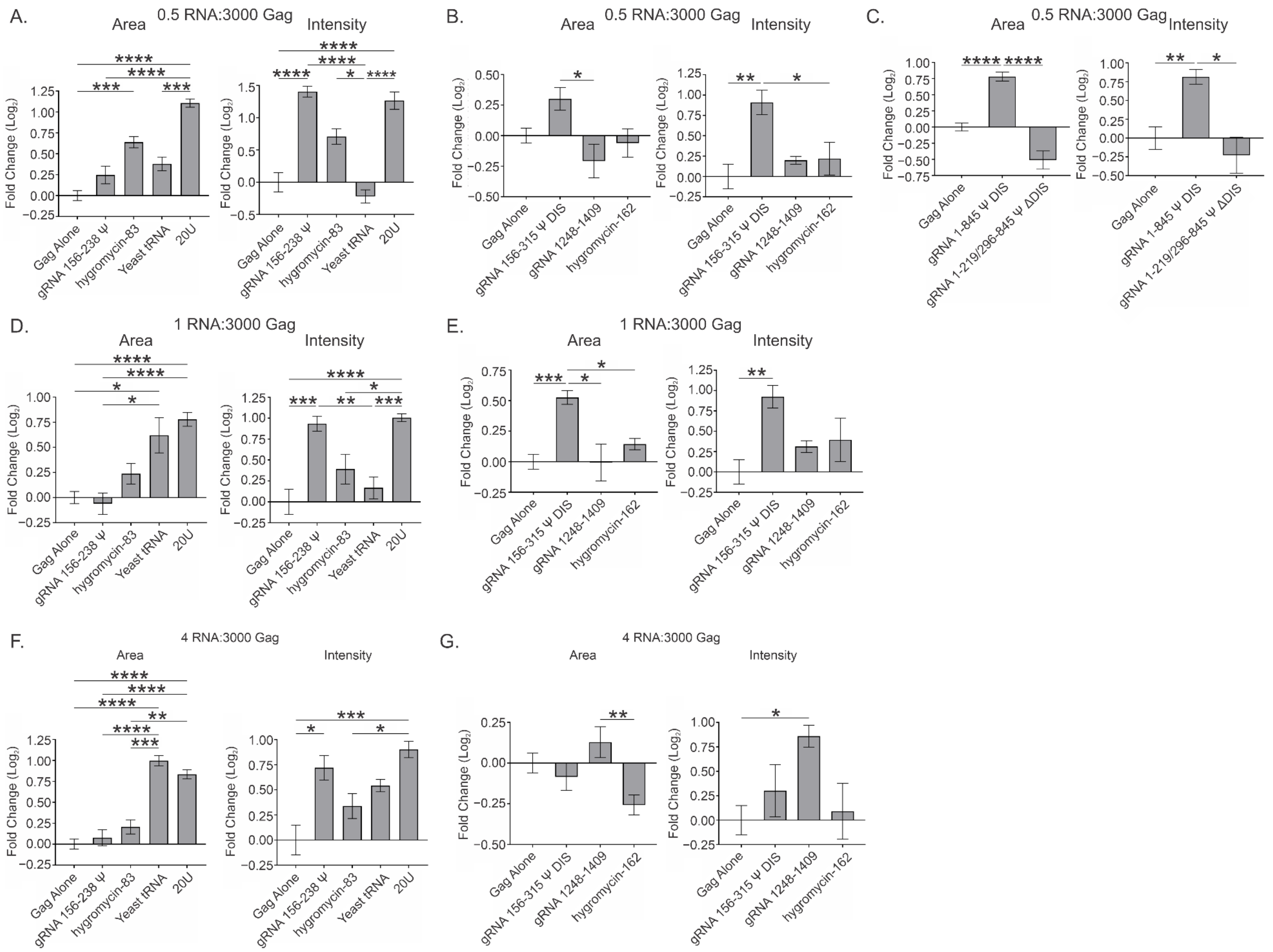

Figure S2. Effect of RNA on 10 μM RSV Gag condensates, 0.5/1/4 RNA:3000 Gag. Fold changes (Log_2) in condensate area and intensity (left and right panels, respectively) for each of three ratios of RNA:3000 Gag (A-C, 0.5 RNA:3000 Gag; D, E, 1 RNA:3000 Gag; F, G, 4 RNA:3000 Gag) and three RNA length groups (A, D, F, short; B, E, G, medium; C, long) are displayed as mean values ± S.E.M. ($n \geq 15$). Statistical significance was determined by Kruskal–Wallis test with Dunn's post-hoc test (****, $p \leq 0.0001$; ***, $p \leq 0.001$; **, $p \leq 0.01$; *, $p \leq 0.05$).

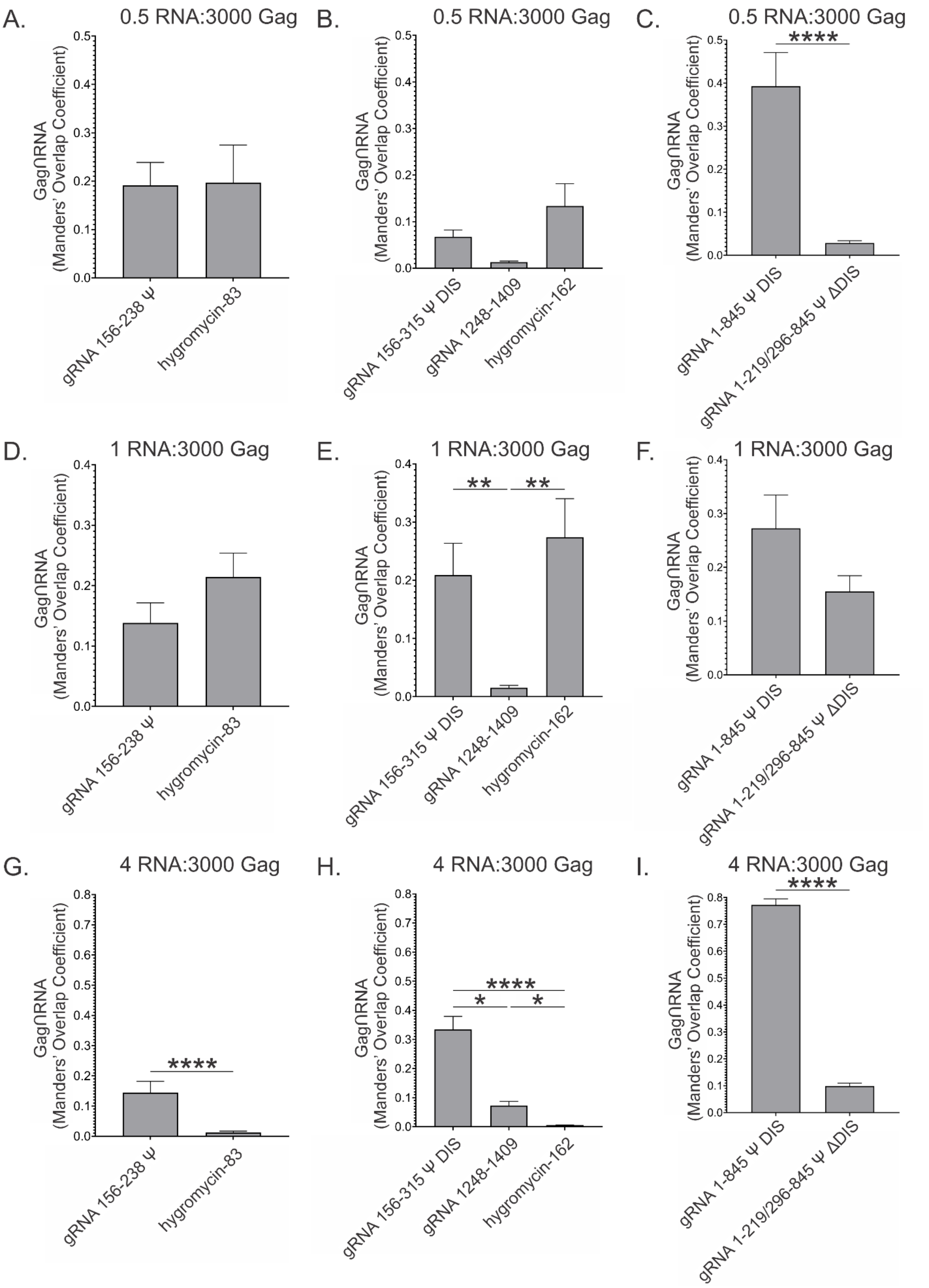

Figure S3. RSV Gag-RNA colocalization within 2.5 μM RSV Gag condensates, 0.5/1/4 RNA:3000 Gag. Manders' Overlap Coefficients for Gag∩RNA for each of three ratios of RNA:3000 Gag (A-C, 0.5 RNA:3000 Gag; D-F, 1 RNA:3000 Gag; G-I, 4 RNA:3000 Gag) and three RNA length groups (A, D, G, short; B, E, H, medium; C, F, I, long) are displayed as mean values ± S.E.M. ($n \geq 9$). Statistical

significance was determined by Mann–Whitney test (A, C, D, F, G, I) or Kruskal–Wallis test with Dunn's post-hoc test (B, E, H) (****, p ≤ 0.0001; **, p ≤ 0.01; *, p ≤ 0.05).

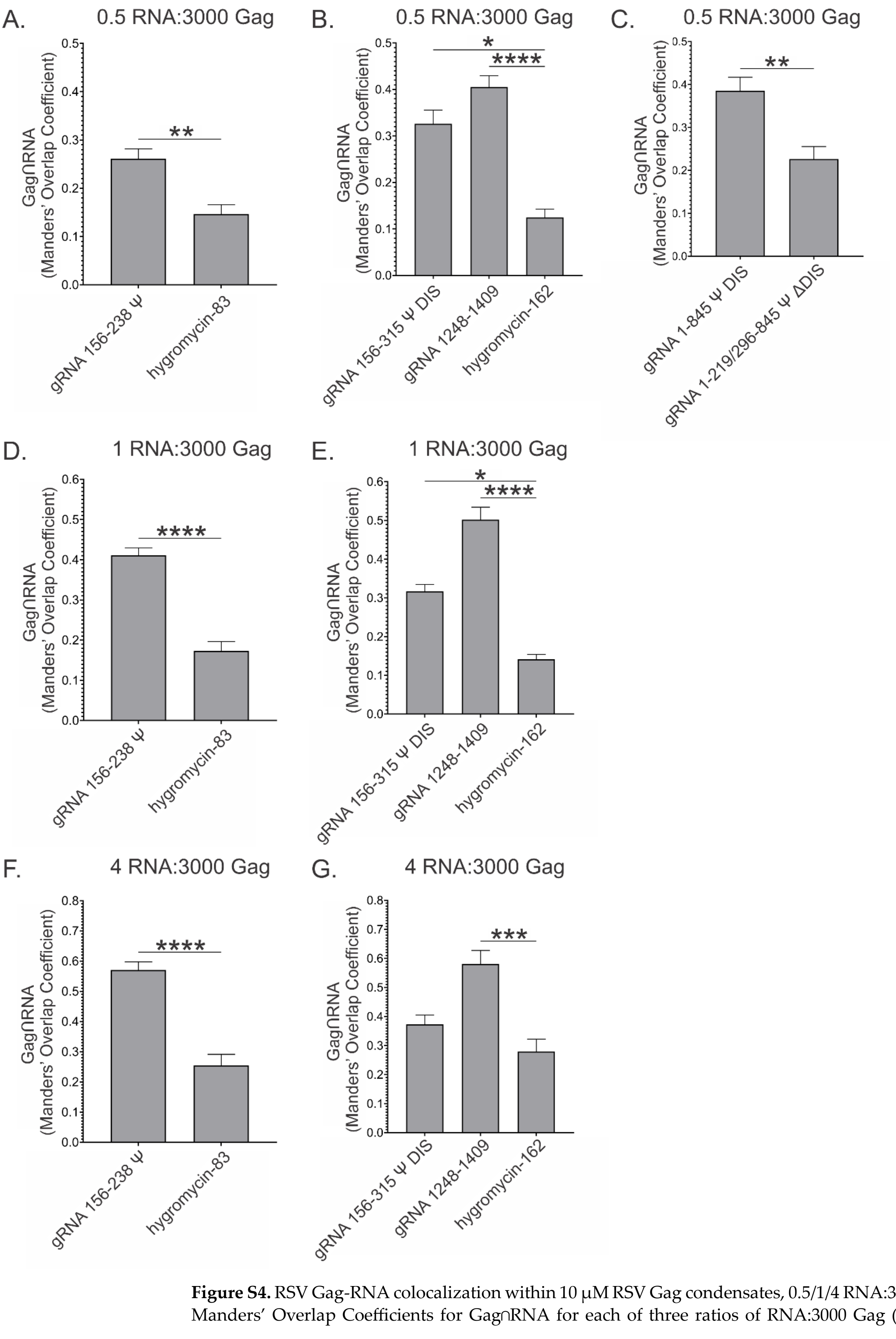

Figure S4. RSV Gag-RNA colocalization within 10 µM RSV Gag condensates, 0.5/1/4 RNA:3000 Gag. Manders' Overlap Coefficients for Gag∩RNA for each of three ratios of RNA:3000 Gag (A-C, 0.5

RNA:3000 Gag; D, E, 1 RNA:3000 Gag; F, G, 4 RNA:3000 Gag) and three RNA length groups (A, D, F, short; B, E, G medium; C, long) are displayed as mean values ± S.E.M. ($n \geq 10$). Statistical significance was determined by Mann–Whitney test (A, C, D, F) or Kruskal–Wallis test with Dunn's post-hoc test (B, E, G) (****, $p \leq 0.0001$; ***, $p \leq 0.001$; **, $p \leq 0.01$; *, $p \leq 0.05$).

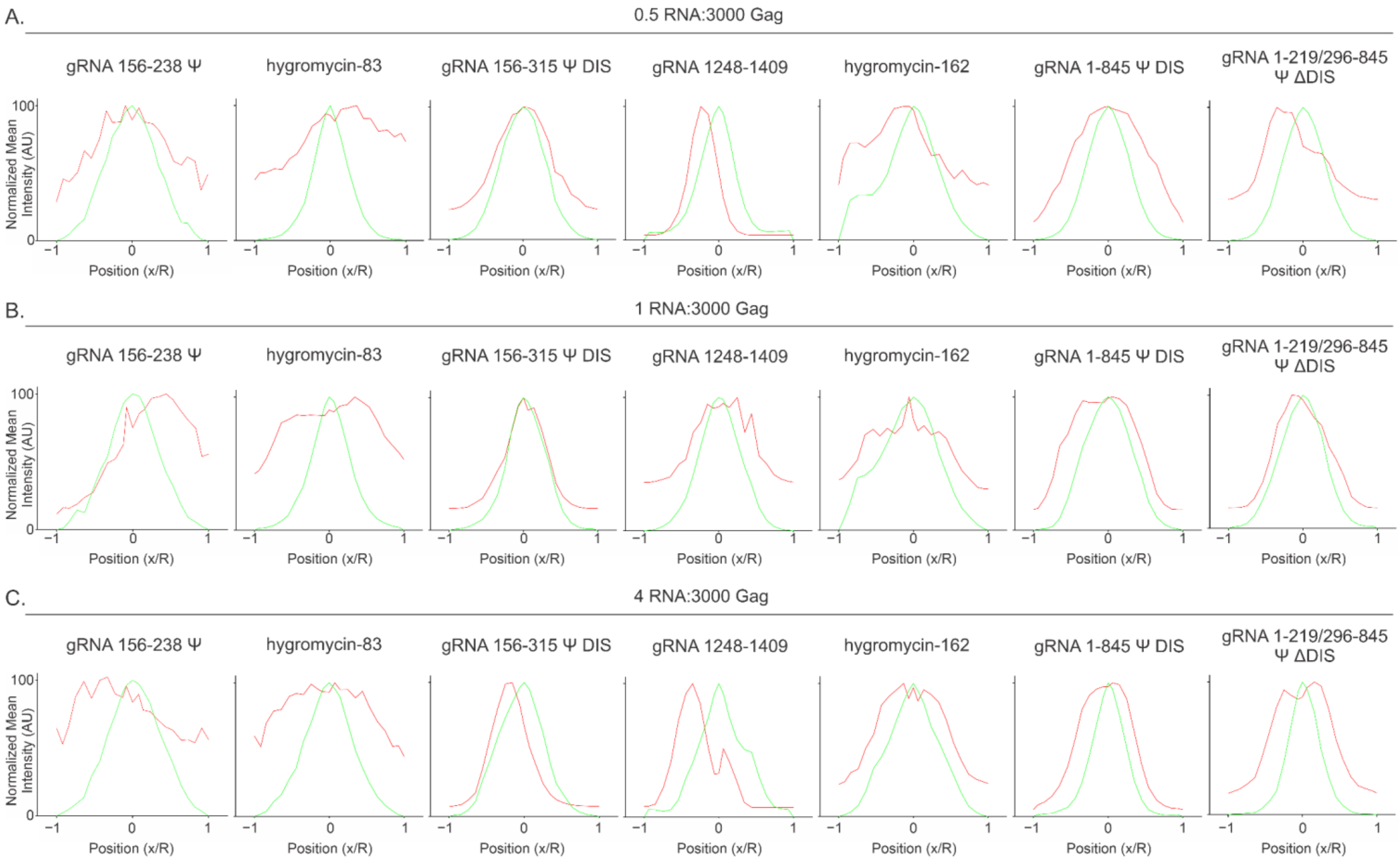

Figure S5. Three-dimensional organization of 2.5 μM RSV Gag-RNA co-condensates, 0.5/1/4 RNA:3000 Gag. Fluorescent signal profiles of RSV Gag (green) and RNA (red) are shown for (A) 0.5 RNA:3000 Gag, (B) 1 RNA:3000 Gag, and (C) 4 RNA:3000 Gag, depicting various levels of signal peak overlap.

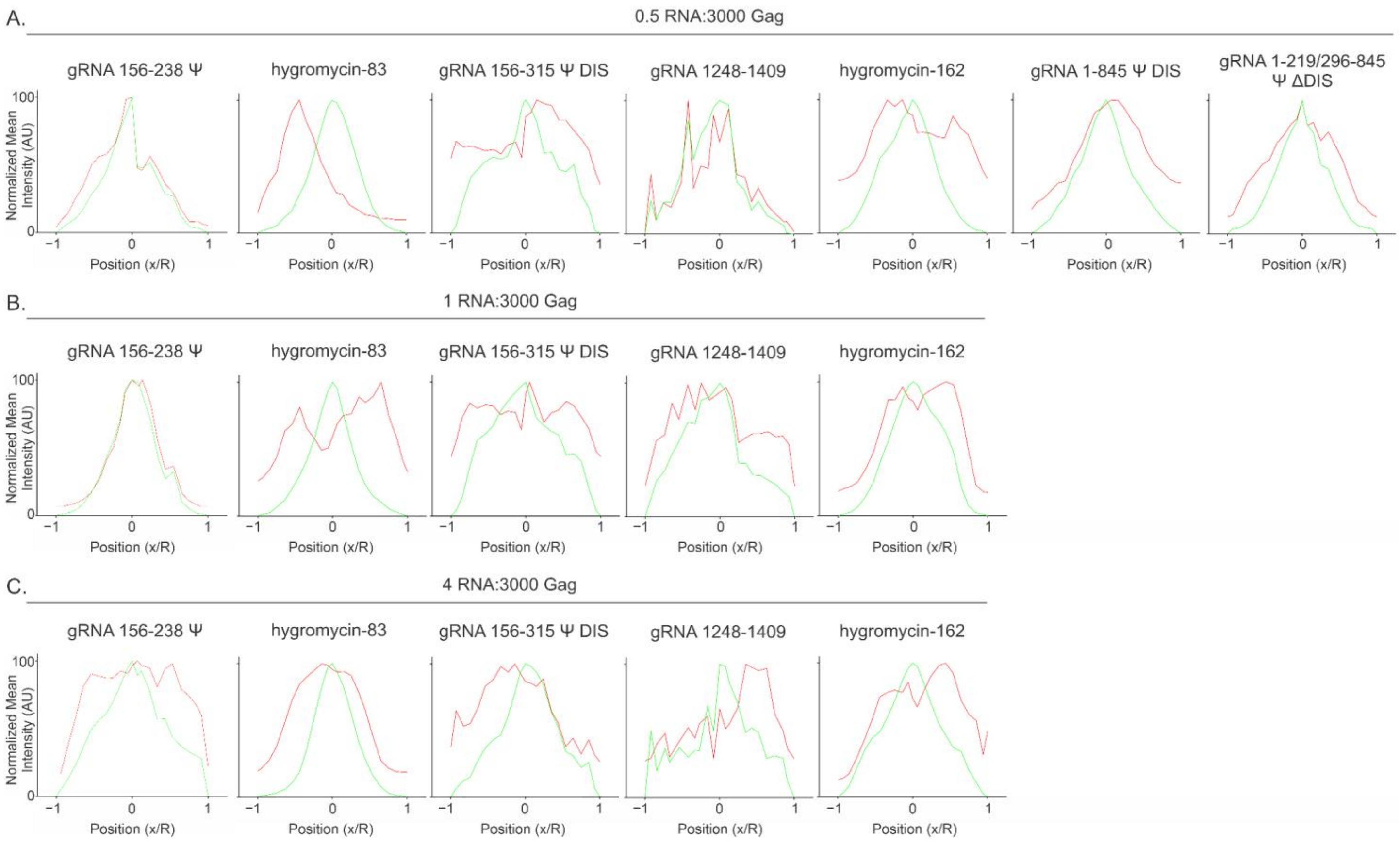

Figure S6. Three-dimensional organization of 10 μM RSV Gag-RNA co-condensates, 0.5/1/4 RNA:3000 Gag. Fluorescent signal profiles of RSV Gag (green) and RNA (red) are shown for (A) 0.5 RNA:3000 Gag, (B) 1 RNA:3000 Gag, and (C) 4 RNA:3000 Gag, depicting various levels of signal peak overlap.

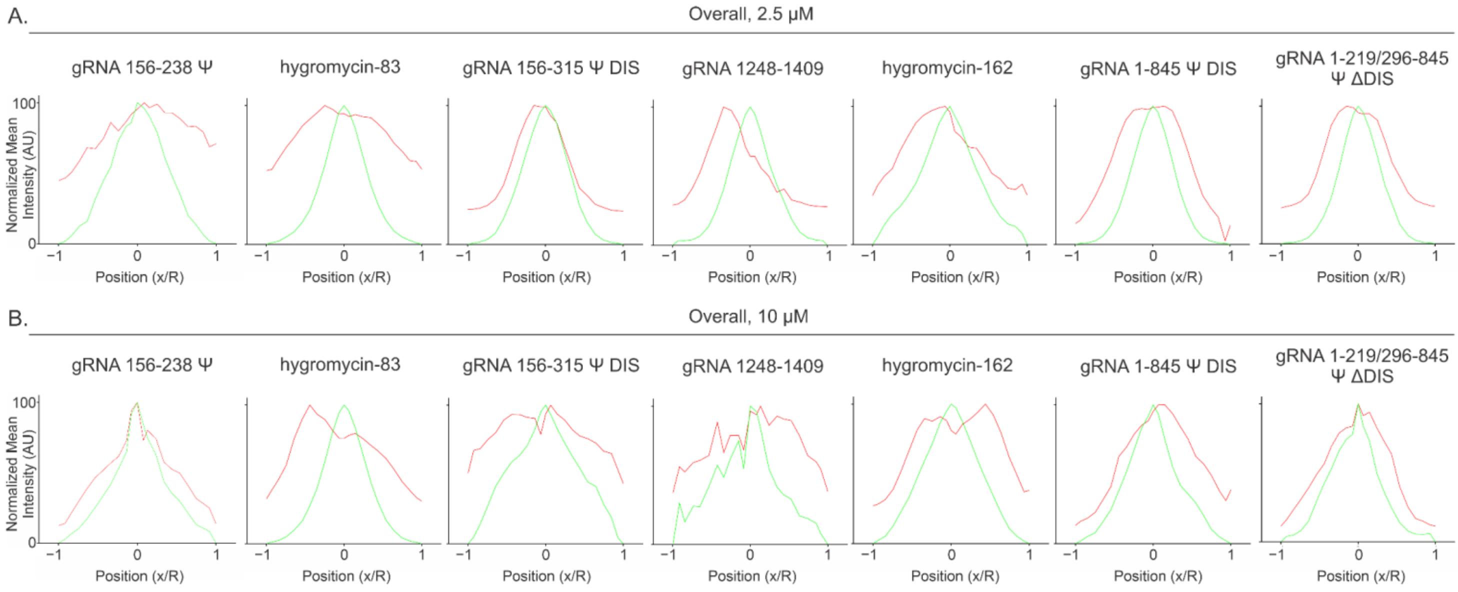

Figure S7. Overall three-dimensional organization of RSV Gag-RNA co-condensates. Overall fluorescent signal profiles of RSV Gag (green) and RNA (red) are shown for 2.5 μM (A) and 10 μM (B) Gag, depicting various levels of signal peak overlap.

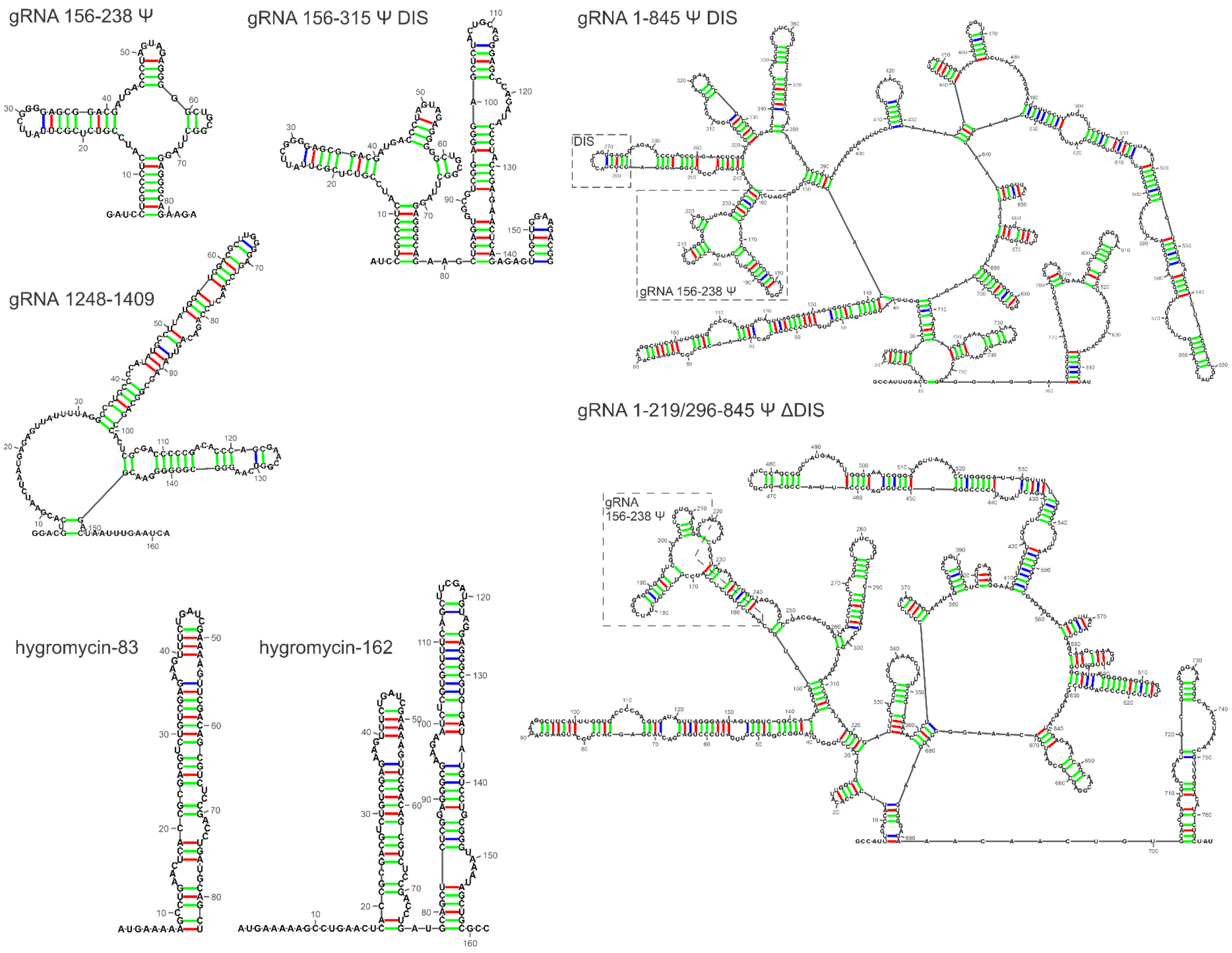

Figure S8. Predicted secondary structure of RNAs. Predicted secondary structures of gRNA 156-238 Ψ, hygromycin-83, gRNA 156-315 Ψ DIS, gRNA 1248-1409, hygromycin-162, gRNA 1-845 Ψ DIS, and gRNA 1-219/296-845 Ψ ΔDIS are displayed. RNA secondary structures were predicted using the RNAstructure web server (Mathews Lab, University of Rochester) and were modeled using RNACanvas. The partition and MaxExpect algorithms were used in conjunction to generate structures with the highest probability of being correct. Regions corresponding to gRNA 156-238 Ψ and the DIS are enclosed in dashed boxes in the gRNA 1-845 Ψ DIS structure, while that of gRNA 156-238 Ψ (156-219, due to the deletion) is similarly boxed in the gRNA 1-219/296-845 Ψ ΔDIS structure. AU (red), CG (green), and GU (blue) base pairs are color coded.

Table S1. 2.5 μM Gag; 0.5 RNA:3000 Gag.

RNA	Area (Log_2 Fold Change vs Gag Alone)	Intensity (Log_2 Fold Change vs Gag Alone)
gRNA 156-238 Ψ	0.738 ± 0.175	1.997 ± 0.221
hygromycin-83	1.810 ± 0.355	1.508 ± 0.183
Yeast tRNA	−0.990 ± 0.609	−2.134 ± 0.663
20U	−2.972 ± 0.964	−3.886 ± 1.062
gRNA 156-315 Ψ DIS	1.866 ± 0.209	2.871 ± 0.291
gRNA 1248-1409	1.668 ± 0.216	0.993 ± 0.235
hygromycin-162	−1.789 ± 0.585	−2.636 ± 0.520
gRNA 1-845 Ψ DIS	1.309 ± 0.248	3.537 ± 0.380
gRNA 1-219/296-845 Ψ ΔDIS	1.442 ± 0.091	1.149 ± 0.095

Table S2. 2.5 μM Gag; 1 RNA:3000 Gag.

RNA	Area (Log$_2$ Fold Change vs Gag Alone)	Intensity (Log$_2$ Fold Change vs Gag Alone)
gRNA 156-238 Ψ	1.035 ± 0.143	1.960 ± 0.146
hygromycin-83	1.520 ± 0.322	1.013 ± 0.368
Yeast tRNA	−0.917 ± 0.387	−1.318 ± 0.373
20U	−1.332 ± 0.344	−2.374 ± 0.372
gRNA 156-315 Ψ DIS	1.193 ± 0.271	2.686 ± 0.253
gRNA 1248-1409	0.001 ± 0.334	−0.728 ± 0.315
hygromycin-162	−2.217 ± 0.692	−2.887 ± 0.623
gRNA 1-845 Ψ DIS	2.174 ± 0.087	3.166 ± 0.235
gRNA 1-219/296-845 Ψ ΔDIS	2.248 ± 0.140	2.345 ± 0.176

Table S3. 2.5 μM Gag; 4 RNA:3000 Gag.

RNA	Area (Log$_2$ Fold Change vs Gag Alone)	Intensity (Log$_2$ Fold Change vs Gag Alone)
gRNA 156-238 Ψ	0.583 ± 0.097	1.748 ± 0.164
hygromycin-83	1.556 ± 0.148	1.094 ± 0.157
Yeast tRNA	0.091 ± 0.185	−0.251 ± 0.197
20U	−1.297 ± 0.786	−2.374 ± 0.847
gRNA 156-315 Ψ DIS	1.586 ± 0.111	2.934 ± 0.153
gRNA 1248-1409	2.576 ± 0.097	2.728 ± 0.130
hygromycin-162	−0.931 ± 0.116	−1.266 ± 0.148
gRNA 1-845 Ψ DIS	2.120 ± 0.085	3.566 ± 0.078
gRNA 1-219/296-845 Ψ ΔDIS	1.692 ± 0.137	1.785 ± 0.198

Table S4. 10 μM Gag; 0.5 RNA:3000 Gag.

RNA	Area (Log$_2$ Fold Change vs Gag Alone)	Intensity (Log$_2$ Fold Change vs Gag Alone)
gRNA 156-238 Ψ	0.246 ± 0.106	1.405 ± 0.083
hygromycin-83	0.638 ± 0.066	0.708 ± 0.121
Yeast tRNA	0.379 ± 0.083	−0.221 ± 0.103
20U	1.106 ± 0.050	1.267 ± 0.134
gRNA 156-315 Ψ DIS	0.301 ± 0.093	0.910 ± 0.150
gRNA 1248-1409	−0.208 ± 0.137	0.198 ± 0.050
hygromycin-162	−0.061 ± 0.115	0.219 ± 0.202
gRNA 1-845 Ψ DIS	0.781 ± 0.071	0.813 ± 0.096
gRNA 1-219/296-845 Ψ ΔDIS	−0.510 ± 0.140	−0.228 ± 0.238

Table S5. 10 μM Gag; 1 RNA:3000 Gag.

RNA	Area (Log$_2$ Fold Change vs Gag Alone)	Intensity (Log$_2$ Fold Change vs Gag Alone)
gRNA 156-238 Ψ	−0.060 ± 0.107	0.934 ± 0.090
hygromycin-83	0.237 ± 0.103	0.391 ± 0.177
Yeast tRNA	0.620 ± 0.176	0.167 ± 0.131
20U	0.778 ± 0.069	1.005 ± 0.047
gRNA 156-315 Ψ DIS	0.525 ± 0.056	0.924 ± 0.139
gRNA 1248-1409	−0.008 ± 0.151	0.312 ± 0.072
hygromycin-162	0.144 ± 0.045	0.394 ± 0.268

gRNA 1-845 Ψ DIS	0.439 ± 0.072	0.462 ± 0.154
gRNA 1-219/296-845 Ψ ΔDIS	−0.374 ± 0.108	−0.220 ± 0.293

Table S6. 10 μM Gag; 4 RNA:3000 Gag.

RNA	Area (Log$_2$ Fold Change vs Gag Alone)	Intensity (Log$_2$ Fold Change vs Gag Alone)
gRNA 156-238 Ψ	0.076 ± 0.095	0.719 ± 0.123
hygromycin-83	0.206 ± 0.084	0.339 ± 0.124
Yeast tRNA	0.996 ± 0.061	0.542 ± 0.062
20U	0.834 ± 0.055	0.902 ± 0.082
gRNA 156-315 Ψ DIS	−0.085 ± 0.082	0.301 ± 0.267
gRNA 1248-1409	0.128 ± 0.095	0.860 ± 0.112
hygromycin-162	−0.258 ± 0.062	0.091 ± 0.285
gRNA 1-845 Ψ DIS	N.D.	N.D.
gRNA 1-219/296-845 Ψ ΔDIS	N.D.	N.D.

N.D., not determined.

Table S7. Composite Factors.

RNA	2.5 μM	10 μM
gRNA 156-238 Ψ	2.547	0.820
hygromycin-83	3.322	0.790
Yeast tRNA	−0.819	0.986
20U	−4.777	1.968
gRNA 156-315 Ψ DIS	4.303	0.937
gRNA 1248-1409	2.396	0.256
hygromycin-162	−4.030	0.195
gRNA 1-845 Ψ DIS	5.335	1.247
gRNA 1-219/296-845 Ψ ΔDIS	3.176	−0.666

Table S8. Gag∩RNA Colocalization.

RNA	2.5 μM Gag			10 μM Gag		
	0.5 RNA: 3000 Gag	1 RNA: 3000 Gag	4 RNA: 3000 Gag	0.5 RNA: 3000 Gag	1 RNA: 3000 Gag	4 RNA: 3000 Gag
gRNA 156-238 Ψ	0.191 ± 0.047	0.138 ± 0.033	0.145 ± 0.037	0.261 ± 0.020	0.411 ± 0.018	0.571 ± 0.027
hygromycin-83	0.197 ± 0.078	0.214 ± 0.039	0.013 ± 0.004	0.146 ± 0.019	0.174 ± 0.023	0.254 ± 0.037
Yeast tRNA	N.D.	N.D.	N.D.	N.D.	N.D.	N.D.
20U	N.D.	N.D.	N.D.	N.D.	N.D.	N.D.
gRNA 156-315 Ψ DIS	0.068 ± 0.014	0.209 ± 0.055	0.334 ± 0.045	0.326 ± 0.030	0.317 ± 0.018	0.373 ± 0.032
gRNA 1248-1409	0.013 ± 0.002	0.015 ± 0.004	0.073 ± 0.014	0.405 ± 0.024	0.502 ± 0.032	0.581 ± 0.046
hygromycin-162	0.133 ± 0.048	0.274 ± 0.067	0.006 ± 0.001	0.125 ± 0.018	0.141 ± 0.013	0.279 ± 0.043
gRNA 1-845 Ψ DIS	0.393 ± 0.078	0.273 ± 0.062	0.772 ± 0.023	0.385 ± 0.032	0.480 ± 0.039	N.D.
gRNA 1-219/296-845 Ψ ΔDIS	0.028 ± 0.006	0.155 ± 0.030	0.099 ± 0.011	0.226 ± 0.029	0.188 ± 0.018	N.D.

N.D., not determined.
